# Adipose-derived Mesenchymal Stromal Cells Modulate Lipid Metabolism and Lipid Droplet Biogenesis via AKT/mTOR –PPARγ Signalling in Macrophages

**DOI:** 10.1038/s41598-019-56835-8

**Published:** 2019-12-30

**Authors:** Luciana Souza-Moreira, Vinicius Cardoso Soares, Suelen da Silva Gomes Dias, Patricia T. Bozza

**Affiliations:** Laboratório de Imunofarmacologia, Instituto Oswaldo Cruz/IOC, Fundação Oswaldo Cruz/FIOCRUZ, Rio de Janeiro, 21045-900 RJ Brazil

**Keywords:** Immunology, Mesenchymal stem cells

## Abstract

Mesenchymal stromal cells (MSCs) are a potential therapy for many chronic inflammatory diseases due to their regenerative, immunologic and anti-inflammatory properties. The two-way dialogue between MSCs and macrophages is crucial to tissue regeneration and repair. Previous research demonstrated that murine adipose-derived MSC conditioned medium (ASCcm) reprograms macrophages to an M2-like phenotype which protects from experimental colitis and sepsis. Here, our focus was to determine the molecular mechanism of lipid droplet biogenesis in macrophages re-educated using ASCcm. Adipose-derived MSC conditioned medium promotes phosphorylation of AKT/mTOR pathway proteins in macrophages. Furthermore, increased expression of PPARγ, lipid droplet biogenesis and PGE_2_ synthesis were observed in M2-like phenotype macrophages (high expression of arginase 1 and elevated IL-10). Treatment with mTOR inhibitor rapamycin or PPARγ inhibitor GW9662 suppressed lipid droplets and PGE_2_ secretion. However, these inhibitors had no effect on arginase-1 expression. Rapamycin, but not GW9662, inhibit IL-10 secretion. In conclusion, we demonstrate major effects of ASCcm to reprogram macrophage immunometabolism through mTOR and PPARγ dependent and independent pathways.

## Introduction

Mesenchymal stem cells (MSCs) are resident mesoderm-derived stromal cells. These cells function as precursors of non-hematopoietic connective tissues which bring about their regenerative therapy potential^1^. However, the clinical interest in MSC cell therapy has gained strength because of their immunomodulatory and anti-inflammatory properties. Murine or human MSCs were effective in preventing survival of graft-versus-host disease, rheumatoid arthritis, experimental autoimmune encephalomyelitis, colitis and sepsis experimental models^2–5^. Indeed, several clinical trials with different tissue sources of MSCs are presently underway due to these findings (ClinicalTrials.gov). However, the dynamic molecular mechanisms involved in the immunoregulatory activity of MSCs are still under exhaustive investigation. MSCs and/or their secretome have been shown to inhibit T cell proliferation, regulate B-cells functions, modulate tolerogenic dendritic cell and induce regulatory T cell^6–9^. Moreover, accumulating evidence demonstrates that adipose tissue-derived MSC (ASC) and conditioned medium from ASC (ASCcm) reprogram macrophages to regulatory/M2-like phenotype. Resulting in protective effects in inflammatory diseases such as experimental colitis and sepsis model^10–12^.

Recent findings place metabolic reprogramming as a key aspect of macrophage activation and function^13^. Indeed, the regulation of immunometabolism is a relevant event during the systemic inflammatory and infection process and it has been connected with evolution theories^14^. It is common knowledge that macrophages engaged in infection responses activate anabolic metabolism similar to those observed during growth/proliferation functions^14^. The proliferation process and metabolic polarization of immune cells are finely tuned by a different molecular mechanism. In macrophages, the high plasticity and phenotype heterogeneity according to environmental signals and stimuli go beyond the classification as anti-inflammatory/regulatory (M2) or inflammatory (M1) functional states^15^. In macrophages activated with LPS (M1), aerobic glycolysis generates energy and is mediated by mTOR/HIF-1α. In contrast, M2 macrophages exhibit mitochondrial metabolism and fatty acid oxidation mediated by mTOR/PPARγ^16–18^. These results indicate that the transition between either glycolytic metabolism or oxidative phosphorylation plays a role in macrophage polarization. Interesting, Vasandan *et al*. showed that MSC-secreted PGE_2_ induce macrophage polarization by modulating the metabolic program^19^.

Nonetheless, few studies have addressed the modulation of lipid droplets in reprogrammed macrophages. Lipids are an essential energy source, critical structural components of cellular membranes and important to cellular signaling. Lipid droplets are lipid-enriched, multifunctional organelles that have multiple roles in physiology and pathological conditions including regulating lipid metabolism and energy homeostasis^20,21^. Indeed, lipid droplets act as essential platforms for immunometabolic regulation, including lipid storage and metabolism, inflammatory lipid mediator production, and signaling pathway compartmentalization. In addition to their function in fat tissues, lipid droplets increase in number and size in cells involved in inflammatory processes. Under different stimulatory conditions, e.g. sterile- or infection-driven inflammation, immune cells like macrophages, neutrophil, and eosinophils exhibit increased lipid droplet biogenesis^22–24^. This process has been associated with increased synthesis and the secretion of inflammatory lipid mediators, such as prostanoids and leukotrienes^25^.

Given the relevance of metabolic, morphological and biochemical alterations of macrophage during the pathophysiology of inflammation response, our hypothesis is that macrophage lipid droplet formation is tightly regulated by ASC; and lipid droplets have key roles in immunometabolic regulations in macrophages. The overall goal of our study was to determine the molecular mechanism of lipid droplet biogenesis and the release of inflammatory lipid mediator in re-educated macrophages by ASC. Here, the mechanisms of lipid droplet biogenesis in M2-like macrophage was demonstrated for the first time.

## Methods

### Animals, cell culture and treatment

C57BL/6 and Balb/c mice (10–15 weeks) were obtained from the Institute of Science and Technology in Biomodels/FIOCRUZ. All animal procedures were approved and performed following the guidelines of the Brazilian Council for care and use of experimental animals (CONCEA). Adipose-derived MSCs (ASCs) were obtained, as previously described^10^. Briefly, perigonadal and inguinal adipose tissues from female Balb/c mice were mechanically and chemically dissociated using a type 1 collagenase 2 mg/mL for 1 h at 37 °C. The cell suspension was filtered using a 100 μm strainer followed by a second filtration step using a 40 μm cell strainer. Cells were centrifuged and resuspended in Mesencult medium (StemCell Technologic). ASCs were maintained at 37 °C in a humidified hypoxic atmosphere containing 5% O_2_, and the conditioned medium was collected between passage 2 and 4. ASC were characterized by morphology and positive expression of CD29 and negative expression of CD45 flow cytometry at passage 3 (Fig. S1). After reaching 80% of confluency, cells were split into 3 T75 flasks and the conditioned medium (ASCcm) was collected after three days in the culture conditions described above. To obtain bone marrow murine macrophages, cells isolated from femur and tibia of male C57Bl/6 mice were cultured using RPMI1640 medium containing 20% heat-inactivated FBS and 30% L929 cell-conditioned medium. Differentiation was performed at 37 °C in a humidified 5% CO_2_ incubator. After seven days, adherent macrophages were harvested and seeded for assays. These macrophages were treated with 50% of ASCcm with the presence or absence of Rapamycin (20 nM) or GW9662 (10 nM) as indicated. Control macrophage (CTRL-MΦ) were treated with 50% of Mesencult medium. The ASC and macrophage culture medium contained 100 U/mL penicillin and 100 µg/mL streptomycin.

### Enzymatic assay

To perform the arginase enzymatic activity assay, after treatment with ASCcm, macrophage cell lysate was collected by using lysis buffer (0.5% Triton-X100, 10 mM Tris, 0.1 mM EDTA, 150 mM NaCl, and 10% glycerol) containing protease inhibitor cocktail (Roche). MnCl_2_, at a final concentration of 1 mM, was added to the lysate; and this solution was heated for 10 min at 56 °C to activate the enzyme. Arginine hydrolysis was conducted by incubating this suspension with 100 μl of 500 mM of L-arginine (pH 9.7) at 37 °C for 40 min. The reaction was stopped using 900 μl of H_2_SO_4_ (96%)/H_3_PO_4_ (85%)/H_2_O (1:3:7, v/v/v) followed by the addition of 40 µL of 9% α-isonitrosopropiophenone (Sigma Aldrich - I3502) that had been dissolved in 100% ethanol. The final solution was incubated for 20 min at 100 °C. Serial dilutions of urea solution were used as the standard. The absorbance was measured at 540 nM to determine the urea concentration in the cell lysate. One unit of arginase activity (U) is defined as the amount of enzyme that catalyzes the formation of 1 µM of urea per min. Protein dosage was determined by Bradford assay and the result was normalized for total protein (mU/µg protein).

### Lipid droplets and immunofluorescence staining

Macrophages were seeded in coverslips and allowed to adhere for 24 h. After treatments, cells were fixed using 3.7% formaldehyde and the lipid droplets were stained with 0.3% Oil Red O (diluted in isopropanol 60%) for 2 min at room temperature. For immunofluorescence staining, after a fixing step, cells were rinsed three times with PBS containing 0.1 M CaCl_2_ and 1 M MgCl_2_ (PBS/CM) and then permeabilized with 0.1% Triton X-100 plus 0.2% BSA in PBS/CM for 10 min (PBS/CM/TB). Cells were stained using rabbit polyclonal anti-PLIN-2 antibody (Proteintech, #15294-1-AP) at 1:1000 dilution for 2 h, followed by a rabbit anti-IgG-Dylight 550 at 1:1000 dilution for 1 h. Lipid droplets were stained with BODIPY493/503 dye (dilution 1:5000 in water) for 5 min. Macrophages were stained with Alexa Fluor™ 594 Phalloidin for 30 min at room temperature (Invitrogen™) for cell morphology analysis. The coverslips were mounted in slides using an antifade mounting medium (VECTASHIELD®). Nuclear recognition was based on DAPI staining (1 µg/mL) for 10 min. Fluorescence was analyzed by fluorescence microscopy with an x100 objective lens (Olympus, Tokyo, Japan) or Confocal Microscopy (Laser scanning microscopy 510 Meta, Zeiss). The numbers of lipid droplets were automatically quantified by ImageJ software analysis or manually counted by light microscopy in 50 consecutive cells.

### SDS-page and western blot

After treatment, macrophages were harvested using ice-cold lysis buffer (1% Triton X-100, 2% SDS, 150 mM de NaCl, 10 mM de Hepes, 2 mM de EDTA plus protease inhibitor cocktail). Cell lysates were heated at 100 °C for 5 min in the presence of Loading buffer (20% β-mercaptoethanol; 370 mM Tris base; 160 μM Bromophenol blue; 6% glycerol; 16% SDS; pH 6.8). 30 μg of protein/sample were resolved by electrophoresis on 10% polyacrylamide SDS-PAGE. After electrophoresis, the separated proteins were transferred to nitrocellulose membranes and incubated in blocking buffer (5% nonfat milk, 50 mM Tris-HCl, 150 mM NaCl, and 0.1% Tween 20). Membranes were probed overnight with the following antibodies: anti-arginase-1 (Santa Cruz Biotechnology, #SC-271430), anti-phospho-AKT (Santa Cruz Biotechnology, #SC-7985), anti-phospho-mTOR (Cell Signaling, #5536), anti-phospho-p70s6k (Cell Signaling, #9205), anti-phospho-4EBP1 (Cell Signaling, #9451), anti-PPARγ (Santa Cruz Biotechnology, #SC-7196 - H100), anti-cPLA_2_ (Santa Cruz Biotechnology. #SC-454), COX-2 (Santa Cruz Biotechnology, #SC-1747) and anti-β-actin (Sigma, #A1978). After the washing steps, they were incubated with IRDye - LICOR or HRP-conjugated secondary. All antibodies were diluted in blocking buffer. The detections were performed by Supersignal Chemiluminescence (GE Healthcare) or by fluorescence using the Odyssey system. The densitometries were analyzed using a software Image Studio Lite Ver 5.2.

### IL-10 and PGE_2_ measure

After 24 h of treatment with ASCcm, M2-like macrophages were rinsed twice with PBS and incubated in fresh medium containing 500 ng/mL of LPS (O127: B8, Sigma) plus 10 ng/mL of IFNy (PeproTech) for 24 h. The IL-10 was quantified by ELISA (murine IL-10 Duo Set, R&D systems) and PGE_2_ by EIA kit (Cayman Chemicals), following manufacturers’ recommended protocols.

### Statistical analysis

Data are expressed as mean ± standard error of the mean (SEM) of three to six independent experiments. The normal distribution was performed using the Shapiro-Wilk test. The paired two-tailed *t*-test was used to evaluate the significance of the two groups. Multiple comparisons among three or more groups were performed by one-way ANOVA followed by Tukey’s multiple comparison test. p values < 0.05 were considered statistically significant when compared to control non-stimulated group or ASCcm treated group.

## Results

### ASCcm polarize and promotes lipid droplet biogenesis in macrophage

**Figure 1:**
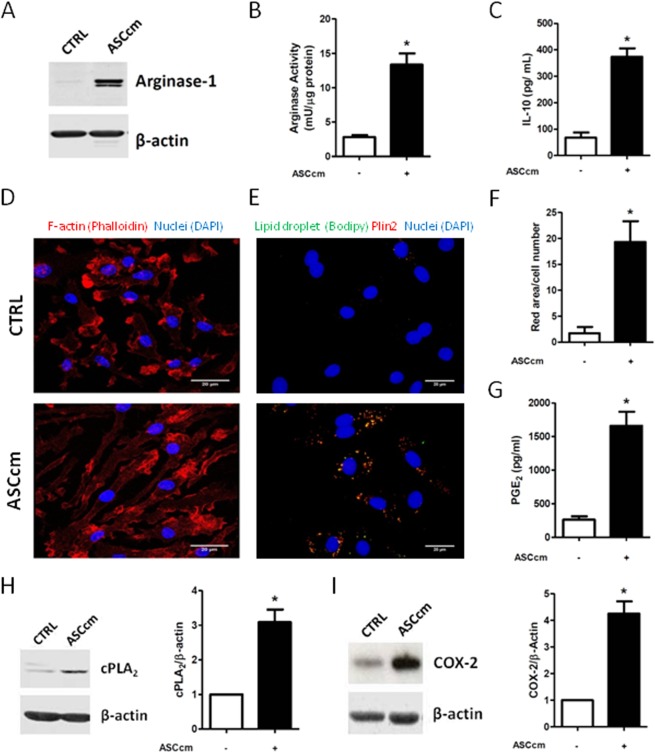
Conditioned medium from Adipose-derived mesenchymal stromal cells (ASCcm) induce macrophage polarization and promote lipid droplet biogenesis. After differentiation with L929 medium, macrophages were seeded and cultured with fresh medium (CTRL) or fresh medium plus ASCcm (50%). After treatment, arginase expression was analyzed by western blot. (**A**) and arginase activity was measured in cell lysates. (**B**) The IL-10 content measured in supernatants by ELISA, after macrophage re-education with ASCcm for 24 h followed by LPS + IFNγ stimulation for an extra 24 h. (**C**) ASCcm induces cellular elongation phenotype in macrophages, as shown by phalloidin staining. (**D**) A significant increase of Lipid droplet number is observed in response to ASCcm. Microscopy images obtained from control (non-treated) or ASCcm- treated macrophage stained with Plin2 (red) and Bodipy (green). The yellow dot represents the merge of Plin2 and Bodipy. (**E**) The images are representative of at least six different experiments. Labeled lipid droplets were quantified by the measurement of fluorescent area per cell using ImageJ software. (**F**) Analysis of PGE_2_ production by macrophage was performed by EIA in the supernatant. (**G**) Analysis of cPLA_2_ and COX-2 in total cell lysates of macrophages by Western blot. (**H**) β-Actin levels were used for control of protein loading. Data are expressed as mean ± SEM of three independent experiment for supernatant dosages and immunofluorescence and six independent experiments for western blot analyses. *p < 0.05 versus non treated cells (CTRL).

Studies from the past suggest that modulation of monocyte/macrophage is the hallmark of MSC therapeutic effects *in vivo* experimental model. Our previous research demonstrated that ASCcm promotes macrophage differentiation towards an M2-like phenotype, which shows high therapeutic capacity in colitis and sepsis experimental models^10^. In order to explore the modulation of lipid mediator on this reprogrammed macrophage, we first confirmed the classical aspects of alternative macrophage phenotype. ASCcm induces high expression and activity of arginase-1 enzyme, a well-known marker for M2/M2-like macrophages (Fig. 1A,B). After stimulation, enhancement in IL-10 production was observed in ASCcm-reprogrammed macrophage (ASC-MΦ) unlike the control macrophage (CTRL-MΦ, non-polarized) (Fig. 1C). In addition, ASC-MΦ showed a distinguished fusiform morphology of cytoplasmic shape compared to CTRL-MΦ, (Fig. 1D). Previously, it was described that cell elongation is a characteristic alteration in an alternative macrophage^26^. These results confirmed the ASCcm programs macrophage toward an alternative M2-like phenotype.

It has been well established that LPS induces M1 macrophages and promotes lipid droplet biogenesis. Here, we described for the first time a remarkable induction of lipid droplets in M2-like macrophages. Those organelles were properly identified by the expression of Plin2 (Fig. 1E). Our findings indicate that ASCcm leads to an increased content of lipid droplets within macrophage’s cytoplasm that appeared as red fluorescent dots in the image (Fig. 1F,G). Lipid droplets are cytoplasmic organelles that compartmentalize PGE_2_ synthesis machinery and final products. In accordance, ASCcm treatment results in the elevated secretion of PGE_2_ by ASC-MΦ (Fig. 1G). Therefore, we evaluated the capability of ASCcm to modulate the expression of crucial enzymes for eicosanoids production. Macrophage treated with ASCcm showed a significant increase in COX-2 and cPLA2-α expression when compared to CTRL-MΦ (Fig. 1H,I). In summary, ASCcm seems to modulate lipid metabolism in macrophages.

### mTOR/PPARγ pathway did not affect macrophage polarization induced by ASCcm

**Figure 2:**
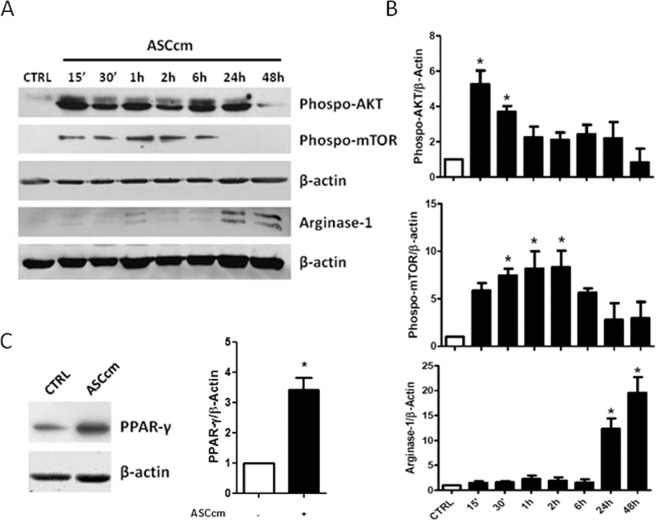
ASCcm activates mTOR/PPARγ signaling in macrophage. Expression of phospo-AKT, phospo-mTOR and arginase-1 by macrophage exposed to ASCcm was analyzed by western blot (**A**) and densitometric data (**B**) for each time point are shown. In (**C**), Immunoblot and densitometric data show increased expression of PPARγ in macrophages exposed to ASCcm for 24 h. The images are representative of at least three different blots and β-actin Data are expressed as mean ± SEM of three independent experiments. *p < 0.05 versus CTRL.

Next, we investigated the mechanisms underlying macrophage polarization in macrophages treated with ASCcm. The mTOR pathway modulates different cells functions, such as survival, proliferation, protein, and lipid synthesis. Further, the regulation of mTOR was showed to be crucial to reprogramming macrophages^27^. The activation of the AKT/mTOR pathway was investigated in different time points after ASCcm treatment. It was concluded that detection of phosphorylated forms of AKT and mTOR was time-dependent. This change was apparent within 15 minutes after the addition of ASCcm and had decreased at 24 hours (Fig. 2A,B). On the other hand, an increase in the arginase-1 expression was only detected at later time points. In addition, our results showed an ASCcm induce expression of PPARγ in comparison to CTRL-MΦ (Fig. 2C). PPARγ is a transcription factor regulated by the mTOR pathway, and it regulates lipid metabolism. Therefore, we used rapamycin, an mTOR inhibitor, to evaluate downstream proteins of this pathway.

**Figure 3:**
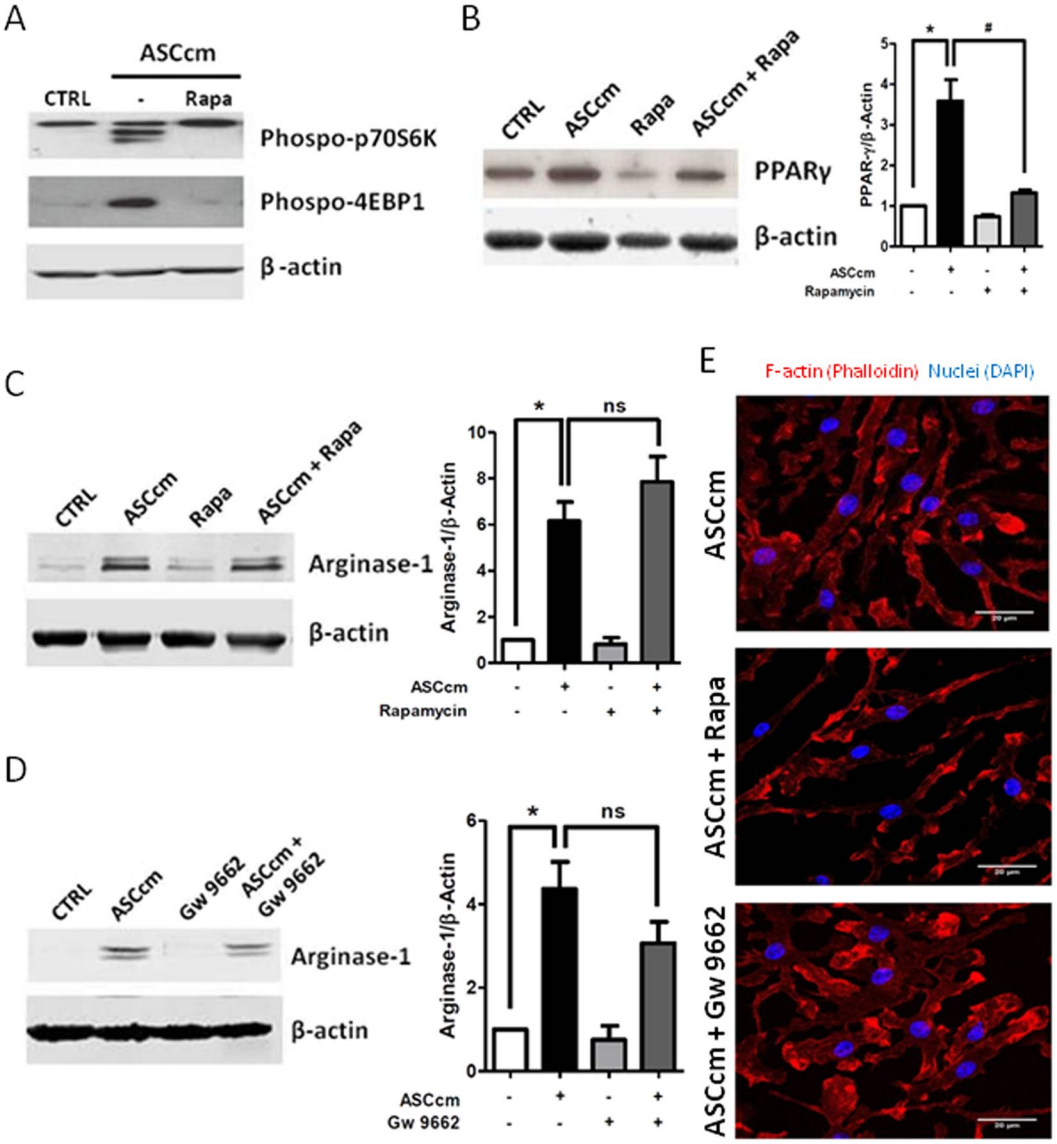
mTOR and PPARγ inhibitors did not affect arginase-1 expression and macrophage elongation morphology induced by ASCcm. Macrophages were pre-treated with mTOR inhibitor Rapamycin (20 nM) or PPARγ inhibitor GW9662 (10 nM), 30 min before treatment with ASCcm (50%). Analysis by western blot of phospo-p70S6K and phospo-4EBP1 after 15 min (**A**) and PPARγ (**B**) after 24 h of treatment with ASCcm. The arginase-1 expression was analyzed in macrophage treated with ASCcm for 24 h in the presence of mTOR (**C**) and PPARγ (**D**) inhibitors. The densitometric data for each inhibitors data set are shown. Representative images of macrophage elongated morphology induced by ASCcm in the presence of rapamycin or GW9662 are shown. (**E**) The images were captured by fluorescent microscopy after F-actin (phalloidin, red) and nuclei (Dapi, blue) staining. Data are expressed as mean ± SEM obtained in three independent experiment for immunofluorescence and five independent experiments for western blot quantification. *p < 0.05 versus non treated cells (CTRL) and ^#^p < 0.05 versus ASCcm treated cells.

**Figure 4:**
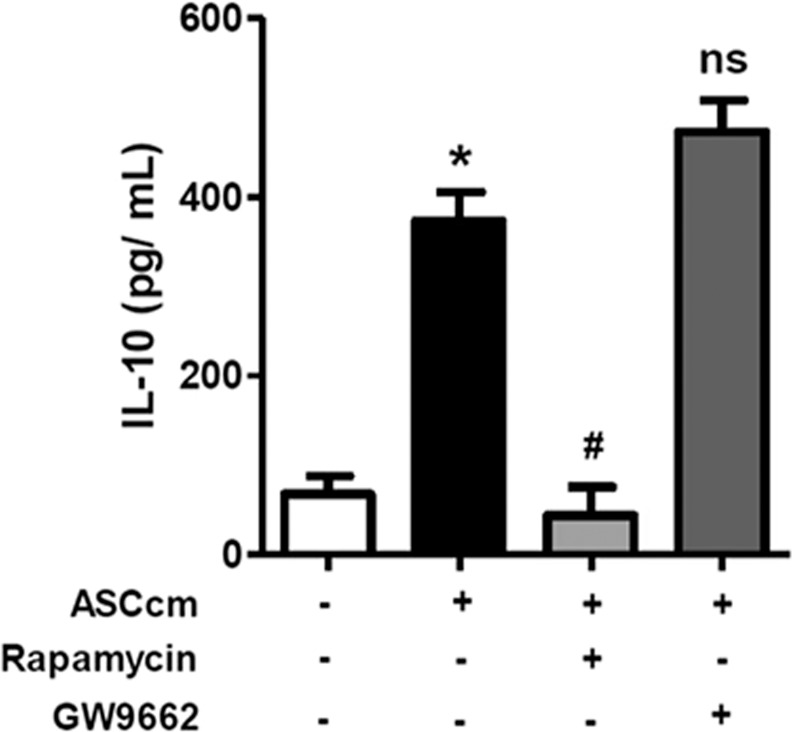
mTOR inhibition, but not PPARγ, suppresses IL-10 secretion induced by ASCcm in macrophage. Macrophages were pre-treated with mTOR inhibitor rapamycin (20 nM) or PPARγ inhibitor GW9662 (10 nM), 30 min before treatment with ASCcm (50%). The IL-10 content measured in supernatants by ELISA, after macrophage re-education with ASCcm for 24 h in presence of the chemical inhibitors, followed by LPS + IFNγ stimulation on fresh medium for 24 h. Data are expressed as mean ± SEM obtained in three independent experiments. *p < 0.05 versus non treated cells (CTRL) and #p < 0.05 versus ASCcm treated cells.

In accordance with the activation of mTOR, the phosphorylation of p70S6k and 4EBP1 was detected after ASCcm treatment. Thus, the rapamycin blocked these effects (Fig. 3A). In addition, the PPARγ expression induced by ASCcm was also significantly inhibited by rapamycin (Fig. 3B). It opened up a possibility of AKT/mTOR/PPARγ pathway to be involved in both lipid droplet biogenesis and macrophage plasticity induced by ASCcm. However, rapamycin and GW9662 (a PPARγ inhibitor) did not affect the macrophage arginase-1 expression and fusiform morphology induced by ASCcm (Fig. 3C–E). Interestingly, rapamycin, but not GW9662, blocked the IL-10 secreted by ASC-MΦ (Fig. 4).

### Lipid droplet biogenesis induced by ASCcm are regulated by mTOR/PPARγ pathway

**Figure 5:**
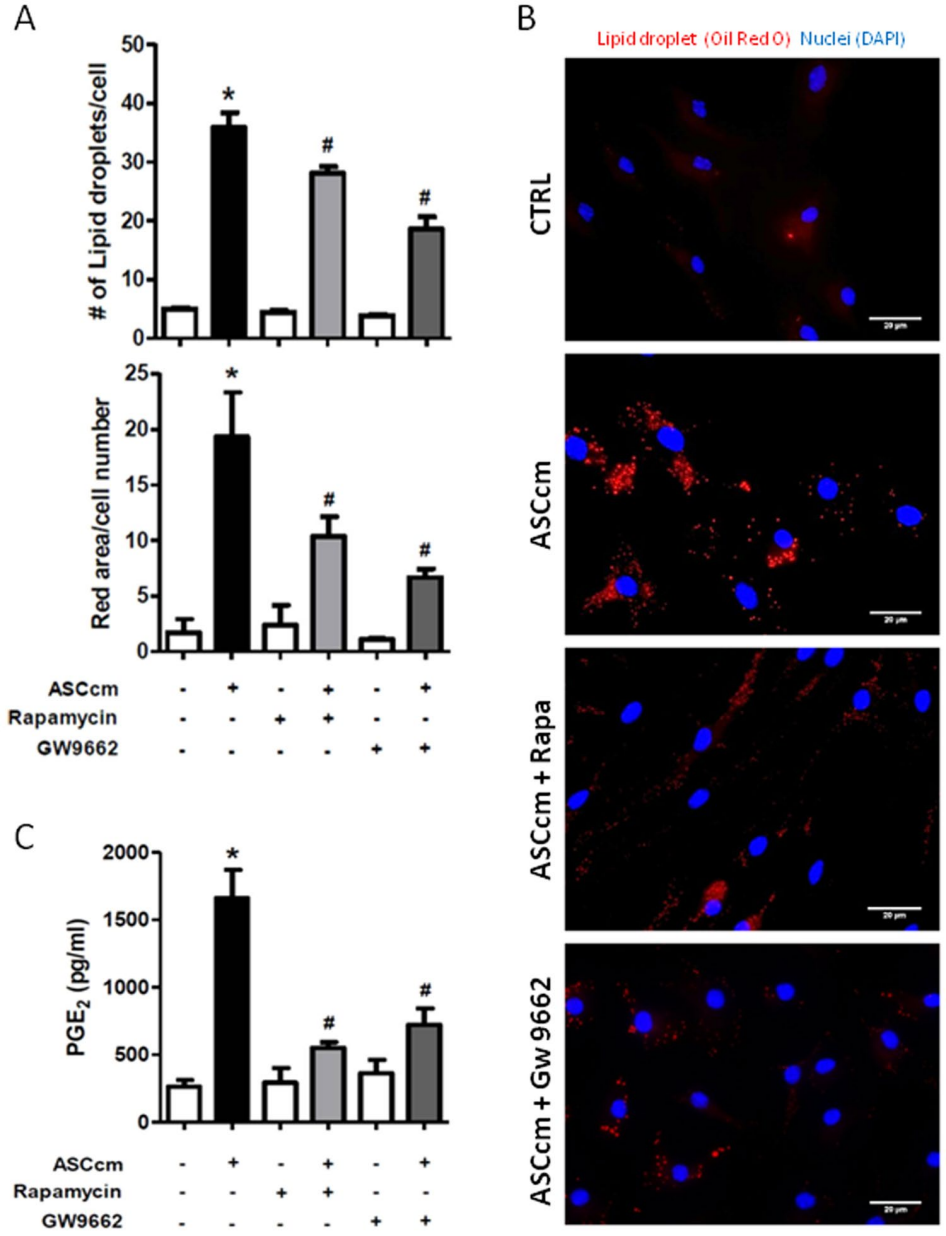
mTOR/PPARγ modulate lipid droplet biogenesis induced by ASCcm in macrophage. Macrophages were pre-treated with mTOR inhibitor rapamycin (20 nM) or PPARγ inhibitor GW9662 (10 nM), 30 min before treatment with ASCcm (50%) for 24 h. In (**A**) lipid droplets were manually enumerated by light microscopy in 50 consecutive cells or evaluated by ImageJ software analysis by the measurement of the fluorescent area. In (**B**) lipid droplets images were captured by fluorescent microscopy after Oil Red O staining. Analysis of PGE_2_ production by macrophage was performed by EIA in the supernatant. (**C**) Data are expressed as mean ± SEM obtained in three independent experiments. *p < 0.05 versus non treated cells (CTRL) and ^#^p < 0.05 versus ASCcm treated cells.

**Figure 6:**
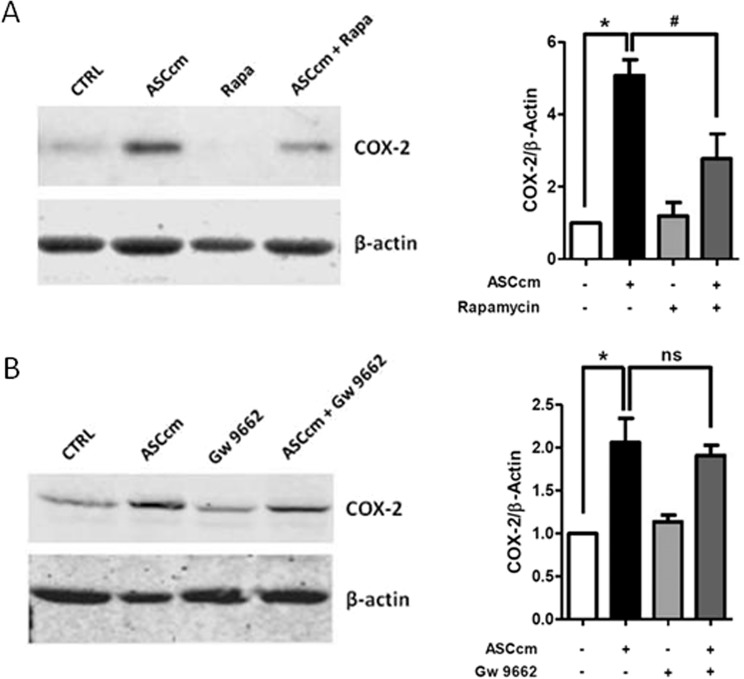
mTOR, but not PPARγ, pathway inhibition affects COX-2 expression induced by ASCcm in macrophage. Macrophages were pre-treated with mTOR inhibitor (**A**) rapamycin (20 nM) or PPARγ inhibitor (**B**) GW9662 (10 nM), 30 min before treatment with ASCcm (50%) for 24 h. Analysis of COX-2 expression was performed in total cell lysates of macrophages by western blot. β-Actin levels were used for control of protein loading. Data are expressed as mean ± SEM from five independent experiments. *p < 0.05 versus non treated cells (CTRL) and ^#^p < 0.05 versus ASCcm treated cells.

Lipid droplets are dynamic organelles controlled by molecular mechanisms that are cell- and stimulus-dependent. Rapamycin and GW9662 treatment were able to reduce lipid droplets induced by ASCcm in macrophage. These effects were showed by the analysis of lipid droplet numbers and the stained area (Fig. 5A,B). Therefore, the production of PGE_2_ was evaluated as a functional marker of these lipid droplets. As expected, rapamycin and GW9662 had a significant impact on PGE_2_ production. This lipid mediator was significantly reduced after inhibition of mTOR and PPARγ protein (Fig. 5C). However, rapamycin treatment, but not GW9662, inhibited COX-2 expression in macrophage treated with ASCcm (Fig. 6A,B). The above observations support the mTOR/PPARγ pathway as a molecular mechanism for lipid droplet biogenesis induced by ASCcm.

## Discussion

**Figure 7:**
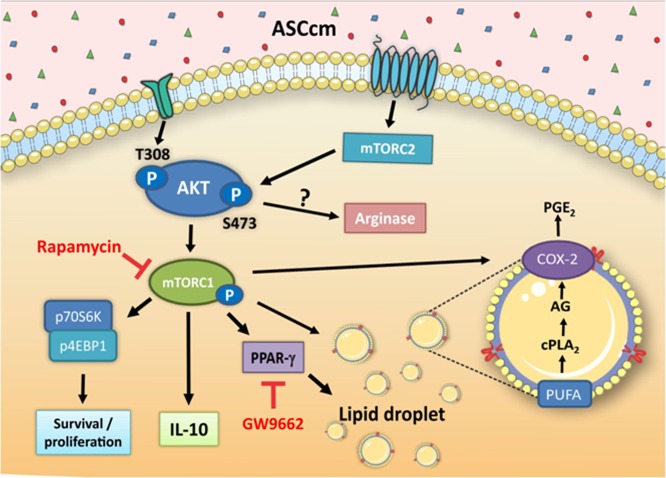
Schematic representation of pathways regulated by ASCcm during macrophage polarization and lipid droplet biogenesis.

Recent advances in understanding the immunomodulatory properties of MSC have provided new insights into their benefits effects on modulating monocytes/macrophage^28^. Thus, it is well-known macrophage plasticity is a crucial component to tissues repair and regeneration. The present study was designed to focus on macrophage polarization and lipid metabolism regulated by ASCcm *in vitro*. We demonstrated that ASCcm induces macrophage polarization to M2-like phenotype accompanied by an increased amount of lipid droplets (Fig. 1). Previously, our research showed that ASCcm converted macrophages into a distinctive phenotype that shares markers with alternatively activated macrophages (M2/M2-like) and regulatory macrophages (Mreg). It includes high expression of arginase I and II, HO-1 and LIGHT and production of nitric oxide and IL-10 upon stimulation, but lack of classical markers of M2 macrophages (ie, Ym1, CCL22 or Fizz1)^10^. Here we demonstrate major effects of ASCcm to reprogram macrophage immunometabolism, leading to increased lipid droplet biogenesis and PGE_2_ production through mTOR and PPARγ dependent pathways (Fig. 7).

It was demonstrated that Balb/c ASC modulates macrophages derived from Balb/c and C57Bl6 and these macrophages have therapeutic effects in two models of inflammatory bowel diseases: 1) TNBS model was performed in Balb/c and 2) DSS model in C57BL/6^10^. In our present study, all the experiments were performed using macrophages derived from bone marrow isolated from male C57Bl/6. Different studies have demonstrated that mesenchymal stem cells from different sources (i.e: tissues, strain) induces macrophage polarization. To better characterize and validate our findings, we performed an experiment using ASCcm isolated from Balb/c and C57BL/6 to compare the induction of lipid droplet biogenesis. ASCcm obtained from Balb/c or C57BL/6 similarly induce increase lipid droplet formation in macrophages (Fig. S2).

The experiments were performed using mesenchymal stem cell obtained from female because they accumulate more adipose tissue than male mice. As such, it allowed us to obtain a reasonable amount of ASC using fewer mice. Although it is clear that there are many genes expressed in a sexually dimorphic manner, the impact of sex in lipid metabolism is explained by multiple factors, including hormones (catecholamine, estrogens, and progesterone), nutritional requirements lipid metabolism in the liver, and systemic metabolism. Therefore, to limit the impact of sex differences on our studies we focused on an *in vitro* model to study the lipid droplet and macrophage polarization. In addition, females were always used to obtain ASC while male mice were used to obtain bone marrow cells.

There is strong evidence that metabolic changes in distinct immune cells play a crucial role during the host immune response against invaders. The paradigm activation of M1 and M2 macrophages have been correlated with a noticeable difference in their metabolic status, by which classically macrophage activation (M1, i.e. activated by LPS plus IFNγ) undergo glycolytic status. In contrast, alternative activation phenotype (M2/Mreg, i.e. activated by IL4 or IL-10) requires mitochondrial β-oxidation of fatty acids^29^. Nonetheless, *in vivo*, it is not clear whether the metabolic and cellular switch is due to repolarization of M1 to M2 (and vice versa) or dependent on new monocytes recruitment. Over the past few years, the immunologist turning the attention to elucidate the signaling pathways involved in metabolic reprogramming in divergent status of immune cells activation. Notwithstanding, it is unclear the role of lipid metabolism in macrophage reprogramming.

Lipid-enriched foam cell macrophages are a hallmark of atherosclerosis. Recently, it was shown that MpOx-LDLs induced lipid droplet accumulation and enhanced anti-inflammatory phenotype in macrophage. MpOX-treated macrophages are more efficient removing tissue debris and apoptotic bodies than M1 and M2 macrophages. Thus, contributes to preventing uncontrolled tissue damage in the atherosclerotic milieu^30^. In this context, MSC inhibits foam cell formation by reducing the uptake of ox-LDL. Furthermore, it decreases dyslipidemia and atherosclerotic lesions size in mice experimental model^31,32^. Supporting this idea, Erpicum *et al*. suggested that administration of MSC prior to renal ischemia/reperfusion attenuates the injury by downregulating fatty acid biosynthesis in kidneys^33^. In contrast, our data showed the unmistakable M2-like polarization induced by adipose-derived MSC is accompanied by increased expression of lipid droplet biogenesis (Fig. 1). Distinct source of MSC could explain the discrepancies between the studies, as bone marrow-derived MSC were used in renal injury and atherosclerosis model. Moreover, we speculate that different sources of MSC (i.e bone marrow, umbilical cord, placenta, etc) as well the source of macrophage (i.e: Kupfer cell, microglia, peritoneal resident macrophage, tumor-associated macrophages, etc) could also impact the modulation of lipid droplets.

Recently, Flaherty *et al*. described in an elegant way that adipose lipid-filled extracellular vesicles (AdEXos) deliver large amounts of triacylglyceride to macrophages. Further, AdExos modulates macrophage differentiation in bone marrow^34^. However, the lipid accumulation is distinct from lipid droplets seen in foam cells. Here we demonstrate the ASCcm induces lipid droplet (confirmed by Plin2 staining; Fig. 1E) and that this event is mediated by mTOR/PPARγ pathway.

mTOR pathway is a nutrient sensor mechanism that regulates cell growth, survival, and metabolic homeostasis. In our experiment, we used 50% of ASCcm and 50% of fresh medium containing serum, to assure macrophage are not under starvation due to possible ASCcm nutrient depletion during conditioning. The control cell (CTRL-MΦ) received 50% of Mesencult media instead of the ASCcm and 50% of fresh medium containing serum. Moreover, conditioned media from L-929 cells (mouse fibroblast-derived from subcutaneous adipose tissue) did not show the same effects as ASCcm (data not shown), suggesting that the effects observed were due to soluble factor secreted by ASC.

Activation of mTOR, a serine/threonine kinase, induces the formation of two distinct protein complexes, known as mTOR Complex 1 (mTORC1) and 2 (mTORC2). Cellular processes downstream of mTORC1 include protein, nucleotide, fatty acid, and lipid synthesis^35^. We demonstrated that ASCcm direct affects the mTORC1 signaling pathway in macrophages by phosphorylation of mTOR and p70S6K (Fig. 2). It was confirmed by rapamycin inhibition of P706K and 4EBP1 phosphorylation, two protein downstream mTORC1 complex (Fig. 3A).

Although we did not have deeply investigated the mTORC2 pathway, our results suggest this pathway could be involved in ASCcm effects over macrophage polarization. First, ASCcm promotes phosphorylation of AKT at Ser473 residue (Fig. 2). The two phosphorylation site of AKT(T308 and S473) are described as functional divisor between mTORC1 and mTORC2 complex^36^. In addition, Rapamycin treatment did not affect arginase-1 expression induced by ASCcm. mTORC2 plays an essential role in M2 macrophages polarization induced by parasitic helminths^27^ and is critical to the metabolic switch induced by IL-4^37^. Our results provide evidence that mTORC1 and mTORC2 cooperate to M2-like macrophage phenotype induced by ASCcm. IL-10 induced by ASCcm was significantly inhibited by rapamycin treatment. This is consistent with the previous report that impaired mTORC1 signals attenuate IL-10 in dendritic cells^38^.

Furthermore, our results showed that ASCcm induces PPARγ expression in macrophages. The activation of AKT/mTOR/PPARγ regulates lipid synthesis and lipid uptake in adipocytes^39,40^. Lipophosphoglycan, a major Leishmania surface glycoconjugate, triggers activation of PPARγ signaling, which leads a rising lipid droplet biogenesis, COX-2, and PGE_2_ production in macrophages^41^. PPARγ is a nuclear receptor associated with glucose and fatty acid metabolism and participates in immune regulation. Rapamycin treatment inhibits partially PPARγ induced by ASCcm and affects the lipid droplet biogenesis (Fig. 5). In accordance, rapamycin treatment reduced fatty acid uptake of adipose tissue explants *in vitro*^39^. The mechanism by which mTOR regulates PPARγ is not completely understood. However, the mTORC1 complex could play a critical role in this pathway, as mTORC2 is less sensitive to rapamycin^35^.

PPARγ regulates COX-2 expression and, consequently PGE_2_ production by PPARγ response element (PPRE) present in the COX-2 gene promoter^42^. However, in our study, PPARγ inhibitor decreases lipid droplet biogenesis and PGE_2_ secretion. But it did not affect COX-2 expression in macrophage re-educated by ASCcm (Fig. 6). This would suggest that PPARγ could affect the expression or the compartmentalization of different enzymes involved in the synthesis and release of inflammatory lipid mediator.

In summary, our data indicate that paracrine factors released by ASC could activate mTORC1 and mTORC2 to stimulate lipid droplet biogenesis and macrophage polarization. However, the specific soluble factors (growth factor, cytokine, lipid mediators) linked to AKT/mTOR activation, macrophage polarization, and lipid droplet biogenesis will be elucidated in future studies. Taken together, our results provide evidence that lipid droplet accumulation could play a dynamic role in macrophage polarization induced by ASCcm. Previous studies showed the effects of human ASC on macrophage polarization^11^. In addition, Zhang *et al*. showed that human and mouse ASC ameliorate the symptoms of acute lung injury in mice^43^. Therefore, we believe that the lipid droplet biogenesis on macrophage could be regulated by human ASC to modulate the inflammatory response and/or tissue repair. However, it needs further investigation to clarify this hypothesis. Nonetheless, further studies are needed to determine the relationship of lipid droplets during the anti-inflammatory/pro-resolution macrophage reprogramming.

## Supplementary information

Supplementary Figures.

Supplementary Dataset.
