# Supplementary material for: Adipose-derived Mesenchymal Stromal Cells Modulate Lipid Metabolism and Lipid Droplet Biogenesis via AKT/mTOR –PPARγ Signalling in Macrophages
Source: Sci Rep. 2019 Dec 30;9:20304. doi: 10.1038/s41598-019-56835-8 (PMC6937267; doi:10.1038/s41598-019-56835-8)
Supplement: Supplementary file 1 — Supplementary Figures. [file 41598_2019_56835_MOESM1_ESM.pdf]

Supplementary Information

Adipose-derived Mesenchymal Stromal Cells Modulate Lipid Metabolism and Lipid Droplet Biogenesis via AKT/mTOR –PPARγ Signalling in Macrophages

Luciana Souza-Moreira[#], Vinicius Cardoso Soares[#], Suelen da Silva Gomes Dias, Patricia T. Bozza*.

Laboratório de Imunofarmacologia, Instituto Oswaldo Cruz/IOC, Fundação Oswaldo Cruz/FIOCRUZ, Rio de Janeiro, 21045-900, RJ, Brazil.

* Address correspondence to Dr. Patricia T. Bozza, pbozza@ioc.fiocruz.br.

#These authors contributed equally to the work

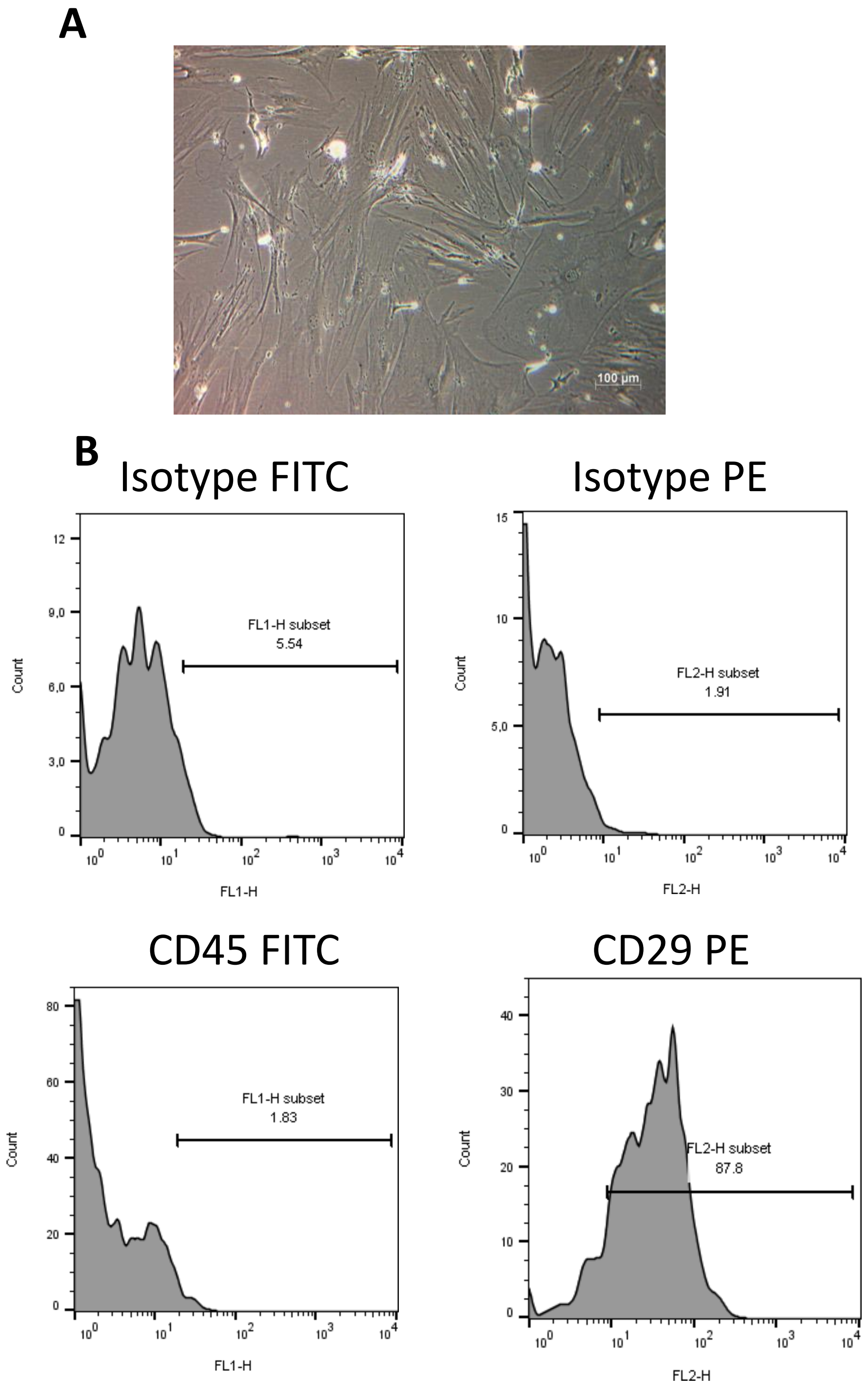

Figure 1S: ASC Characterization. ASCs were isolated from perigonadal and inguinal adipose tissues and maintained at 37 °C in a humidified hypoxic atmosphere. Cells were characterized by adherent cell morphology(A) and flow cytometry (B) at passage 3. Representative flow cytometry plots are showing the positive expression of CD29 and the negative expression of CD45.

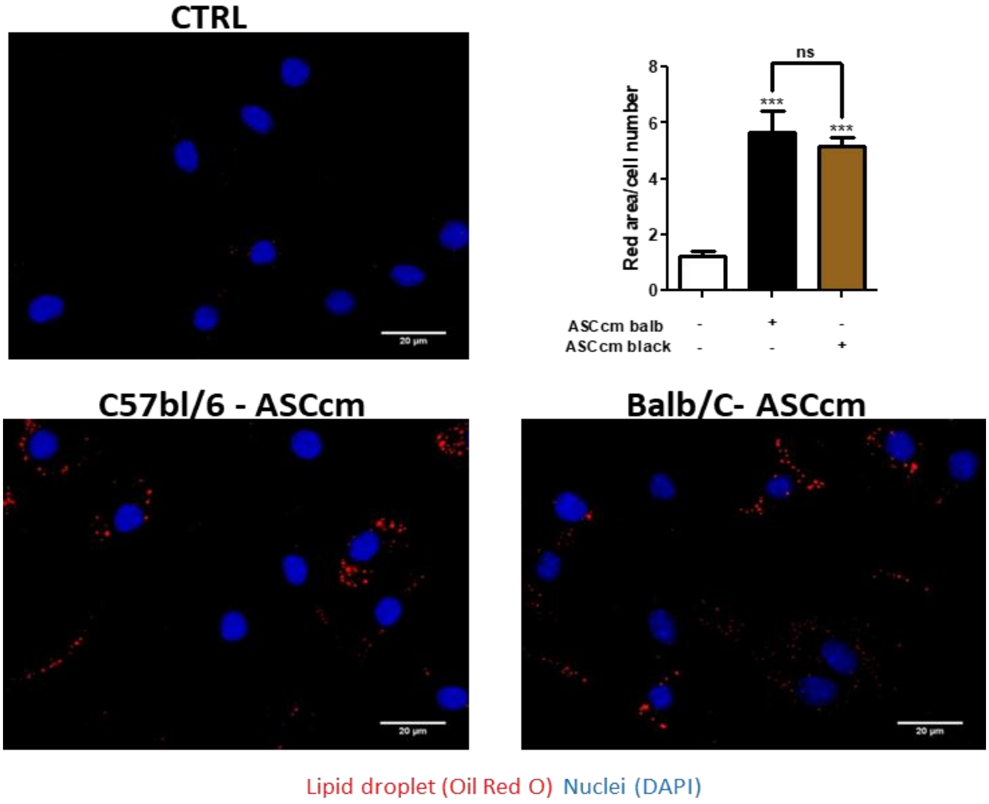

Figure 2S: Conditioned medium from Balb/c- ASC or C57BL/6-ASC promote lipid droplet biogenesis in bone marrow derived macrophage from C57BL/6. Representative images show non-treated (CTRL) or ASCcm- treated macrophage stained with Oil Red O (lipid droplets; red) and Dapi (nuclei; blue). Labeled lipid droplets were quantified by the measurement of fluorescent area per cell using ImageJ software.
