# Supplementary material for: Adipose-derived Mesenchymal Stromal Cells Modulate Lipid Metabolism and Lipid Droplet Biogenesis via AKT/mTOR –PPARγ Signalling in Macrophages
Source: Sci Rep. 2019 Dec 30;9:20304. doi: 10.1038/s41598-019-56835-8 (PMC6937267; doi:10.1038/s41598-019-56835-8)
Supplement: Supplementary file 2 — Supplementary Dataset. [file 41598_2019_56835_MOESM2_ESM.pdf]

Supplementary Information File

Adipose-derived Mesenchymal Stromal Cells Modulate Lipid Metabolism and Lipid Droplet Biogenesis
via AKT/mTOR –PPARγ Signalling in Macrophages

Luciana Souza-Moreira, Vinicius Cardoso Soares, Suelen da Silva Gomes Dias, Patricia T. Bozza.

Full-length blots used in the article are indicated according to each figure. Red squares represent the images selected as a representative blot, each number in the images are the replicate of the experiment used to quantified the blots.

Figure 1A

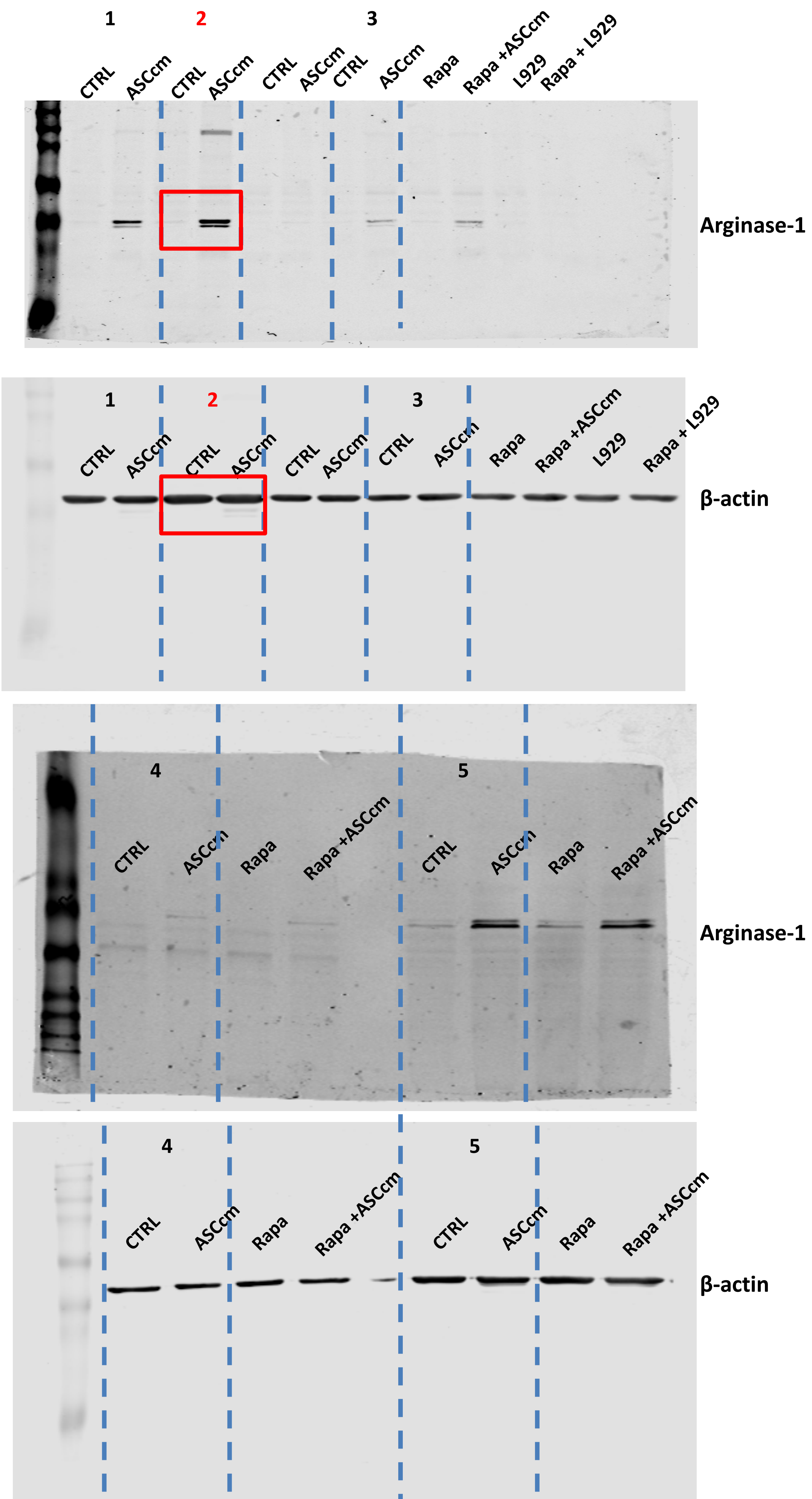

1
2
3
CTRL
ASCcm
CTRL
ASCcm
CTRL
ASCcm
CTRL
ASCcm
Rapa
Rapa +ASCcm
L929
Rapa + L929
Arginase-1
1
2
3
CTRL
ASCcm
CTRL
ASCcm
CTRL
ASCcm
CTRL
ASCcm
Rapa
Rapa +ASCcm
L929
Rapa + L929
β-actin
4
5
CTRL
ASCcm
Rapa
Rapa +ASCcm
CTRL
ASCcm
Rapa
Rapa +ASCcm
Arginase-1
4
5
CTRL
ASCcm
Rapa
Rapa +ASCcm
CTRL
ASCcm
Rapa
Rapa +ASCcm
β-actin

Figure 1H

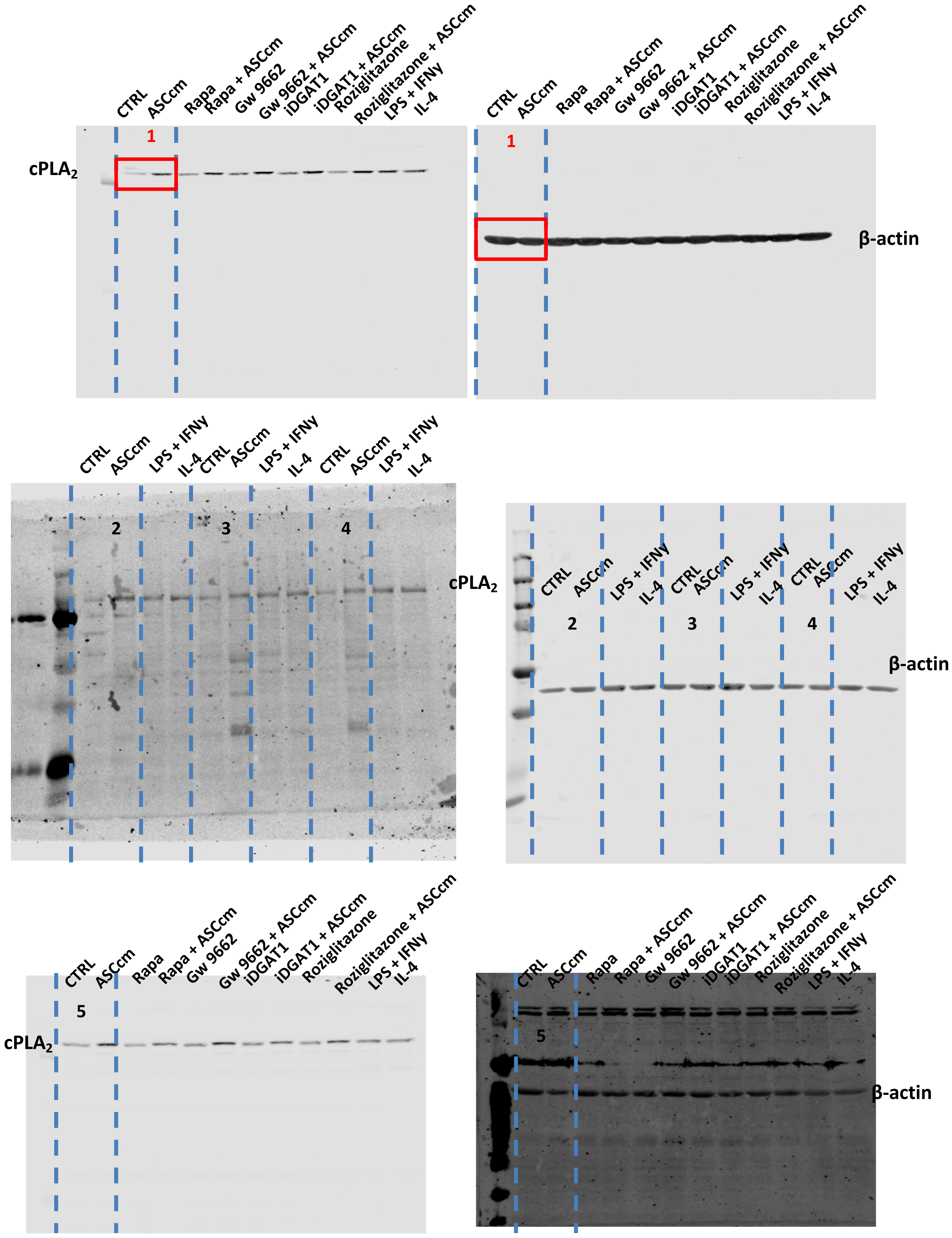

CTRL
ASCcm
Rapa
Rapa + ASCcm
Gw 9662
Gw 9662 + ASCcm
iDGAT1
iDGAT1 + ASCcm
Rozyglitazone
Rozyglitazone + ASCcm
LPS + IFNγ
IL-4
cPLA2
β-actin
1
2
3
4
5

Figure 1I

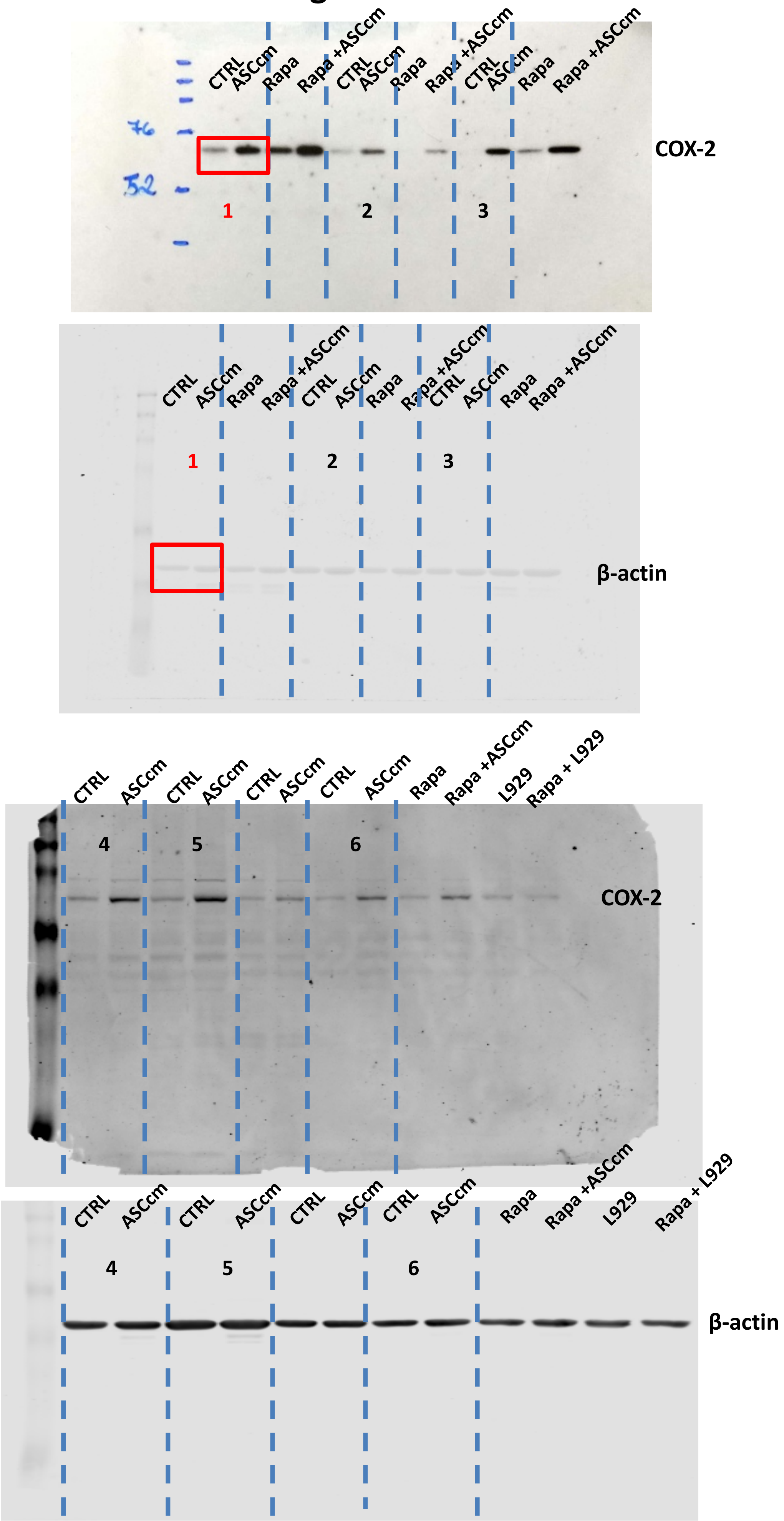
CTRL
ASCcm
Rapa
Rapa +ASCcm
76
52
1
2
3
COX-2
β-actin
4
5
6
L929
Rapa + L929

Figure 2A – p-AKT

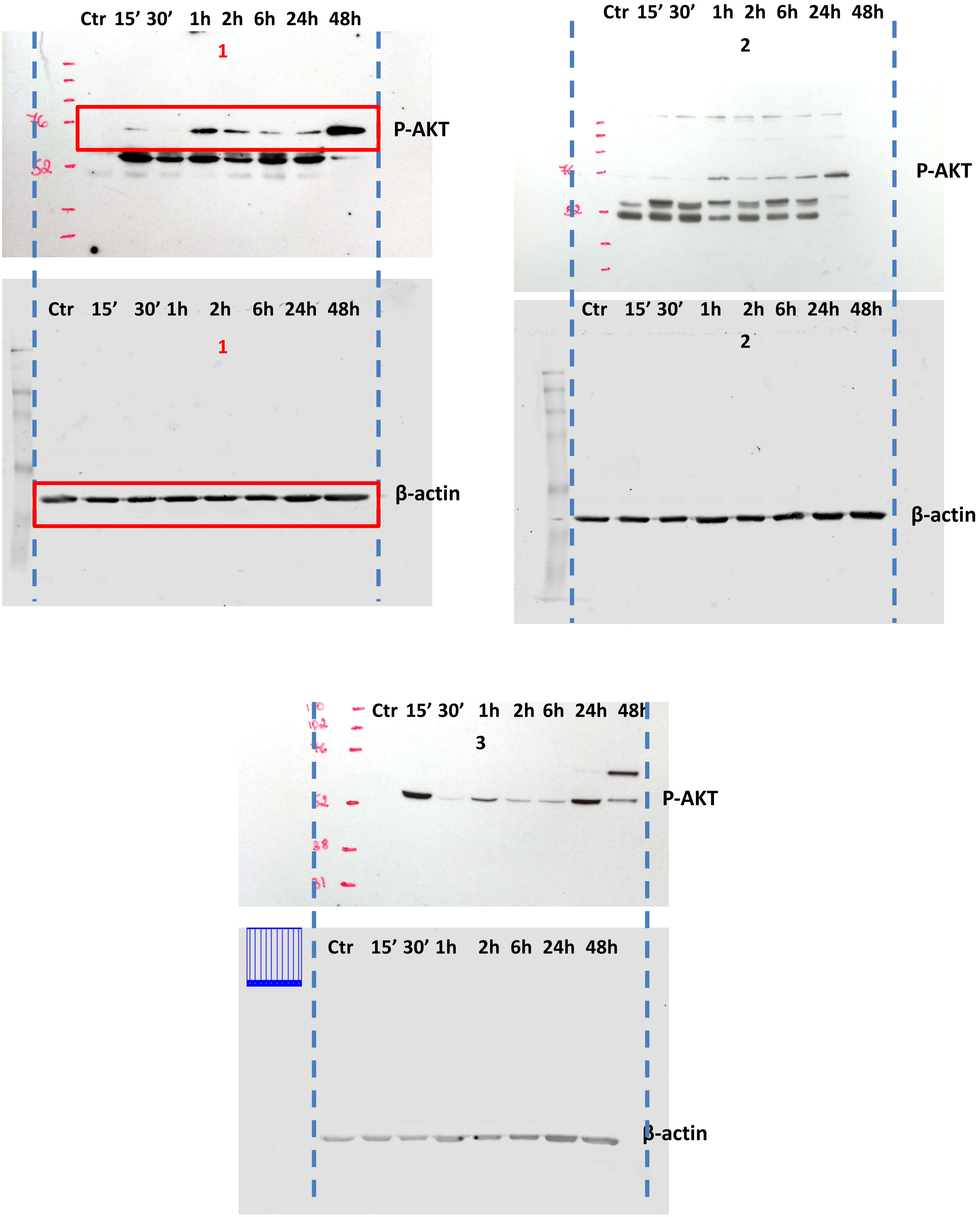

Figure 2A – p-mTOR

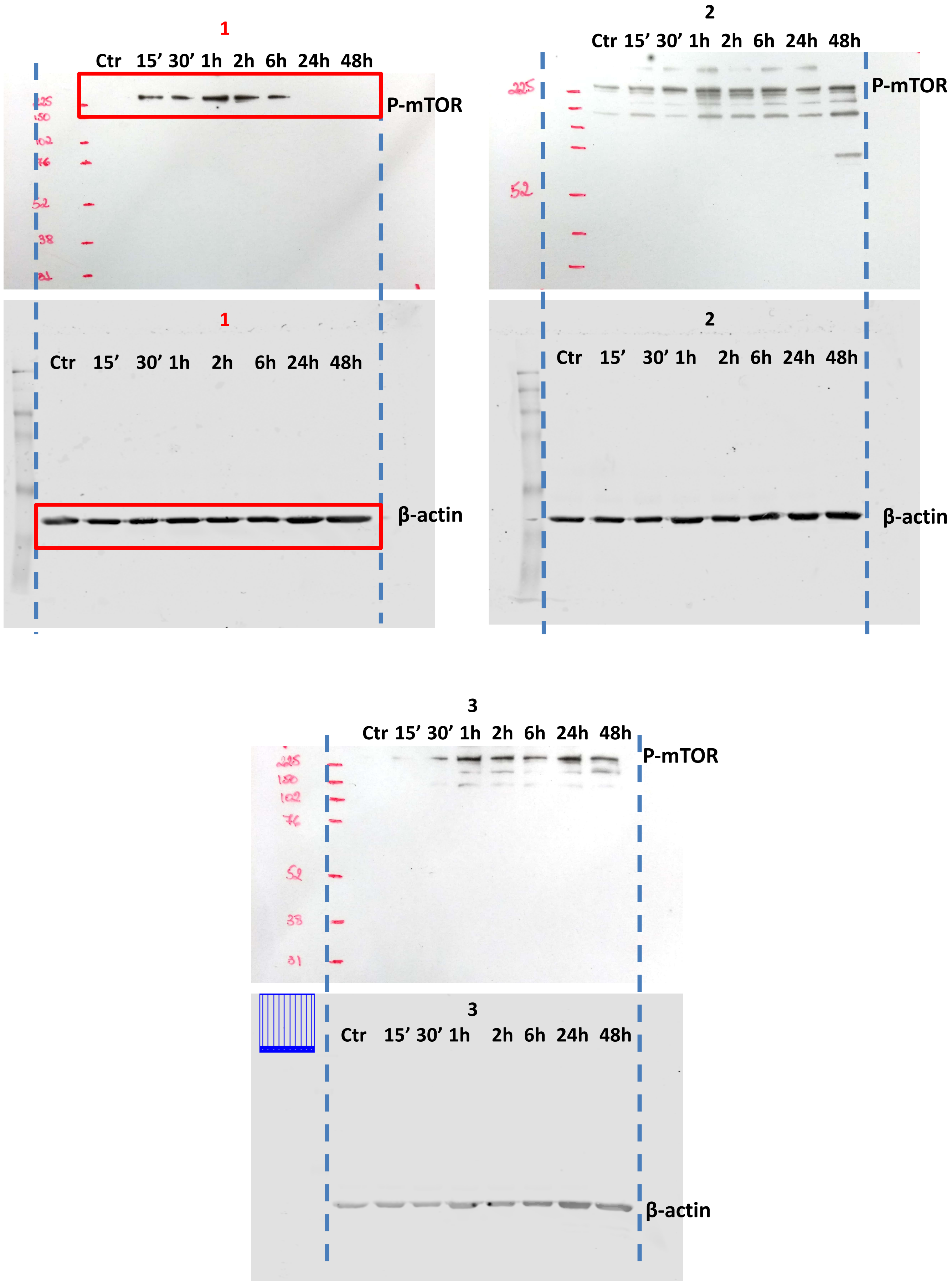

Figure 2A - Arginase

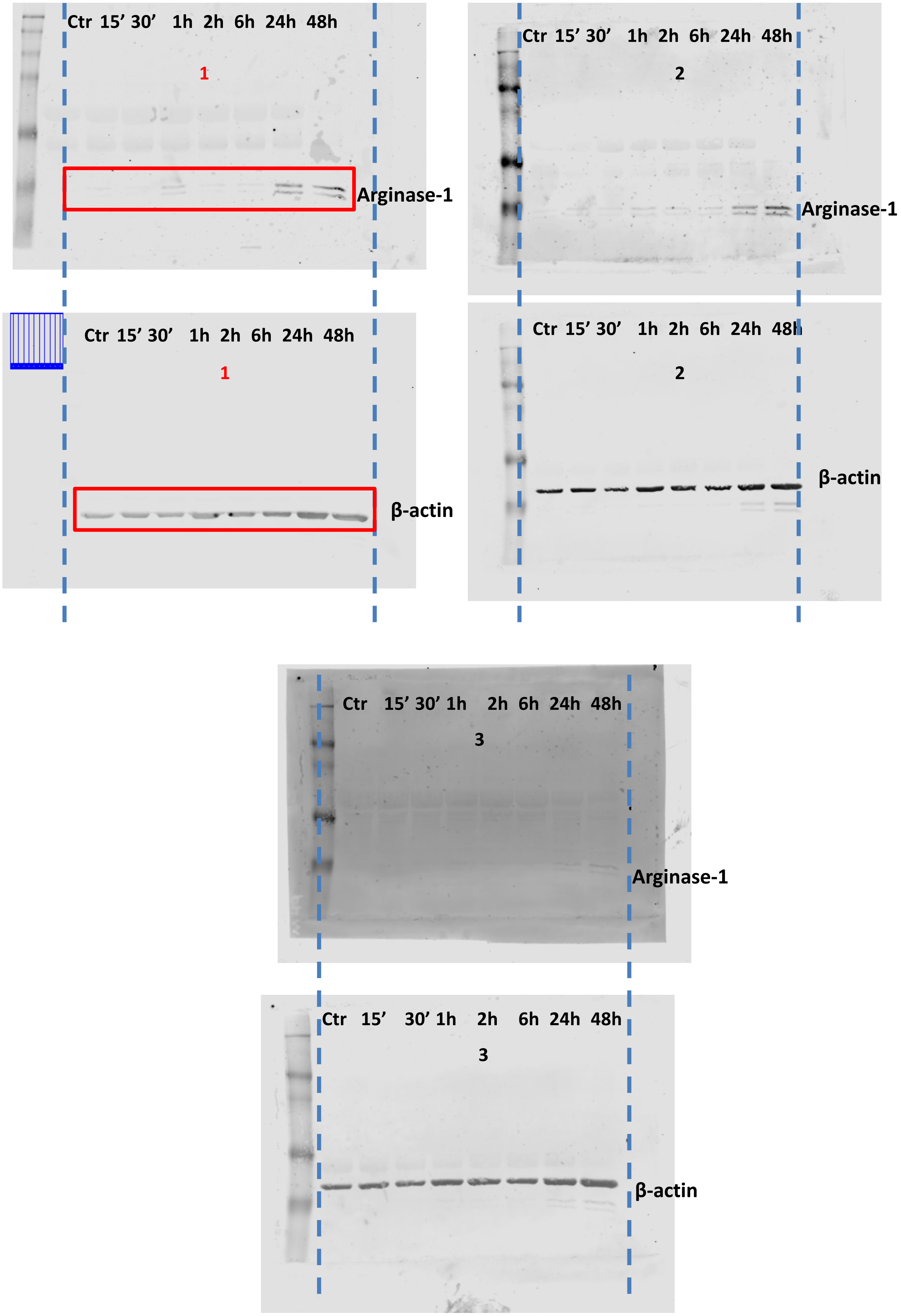

Figure 2C

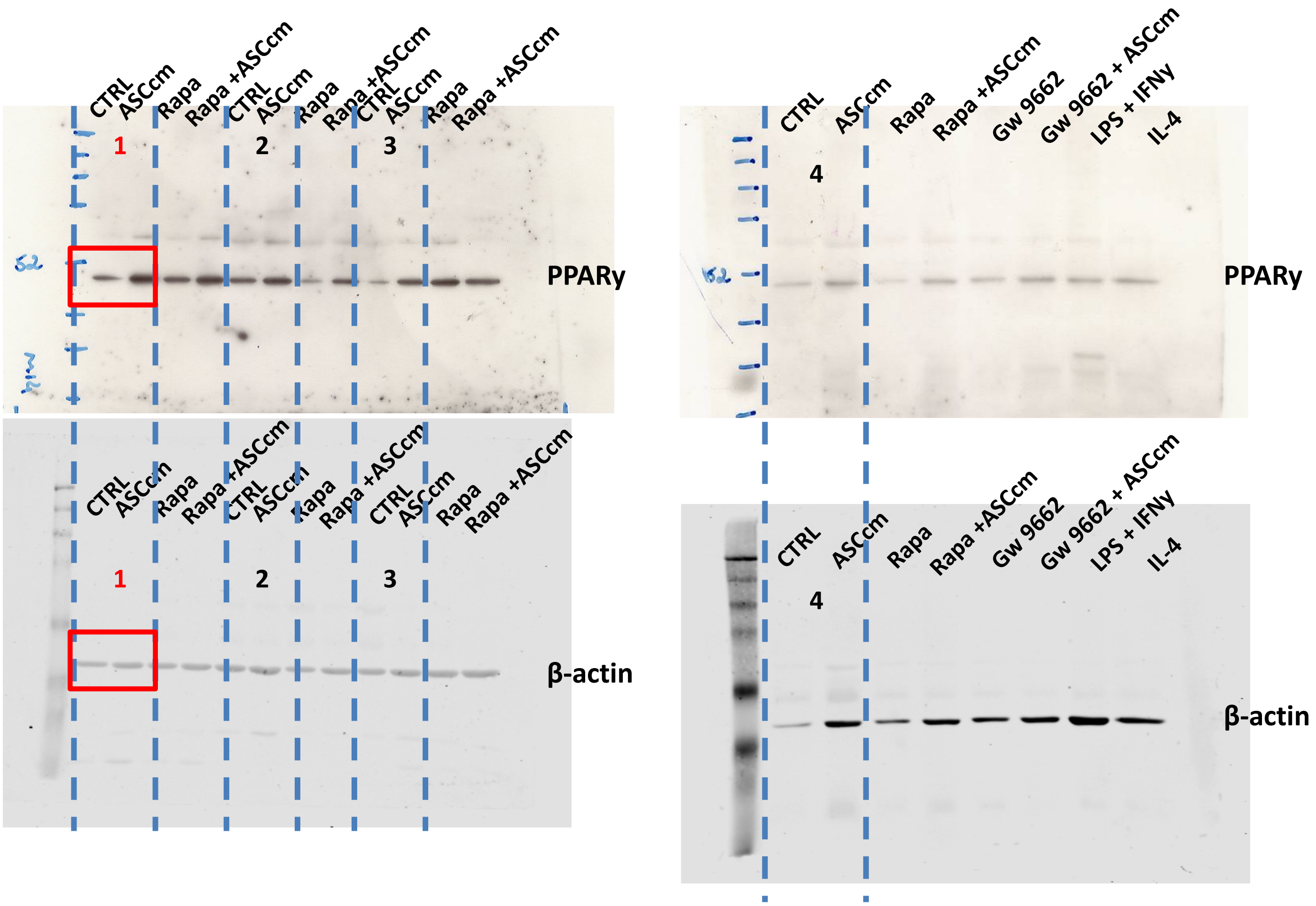
CTRL
ASCcm
Rapa
Rapa +ASCcm
1
2
3
PPARγ
β-actin
4
Gw 9662
Gw 9662 + ASCcm
LPS + IFNγ
IL-4

Figure 3A - p-70s6k

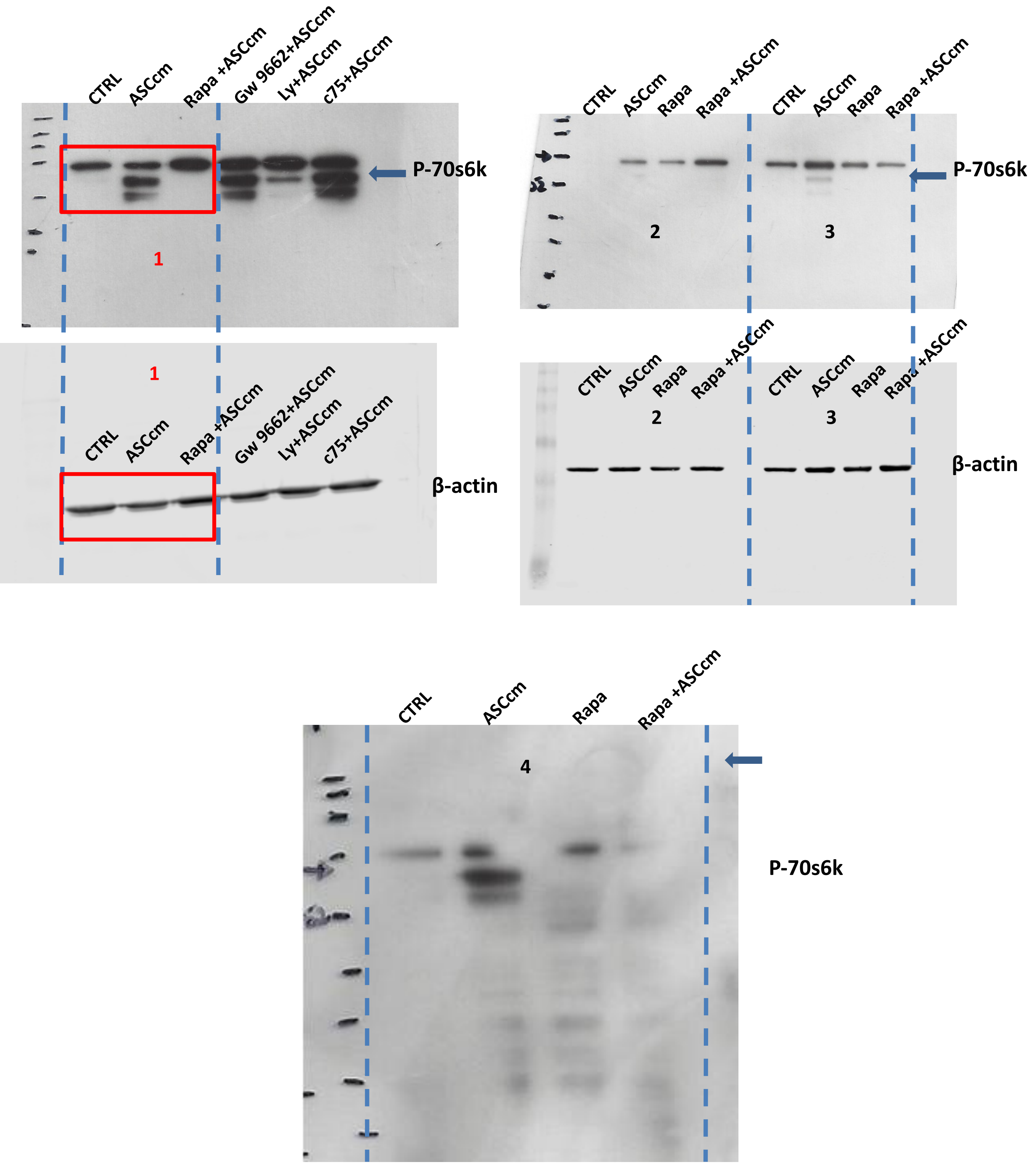

Figure 3A – p-4EBP1

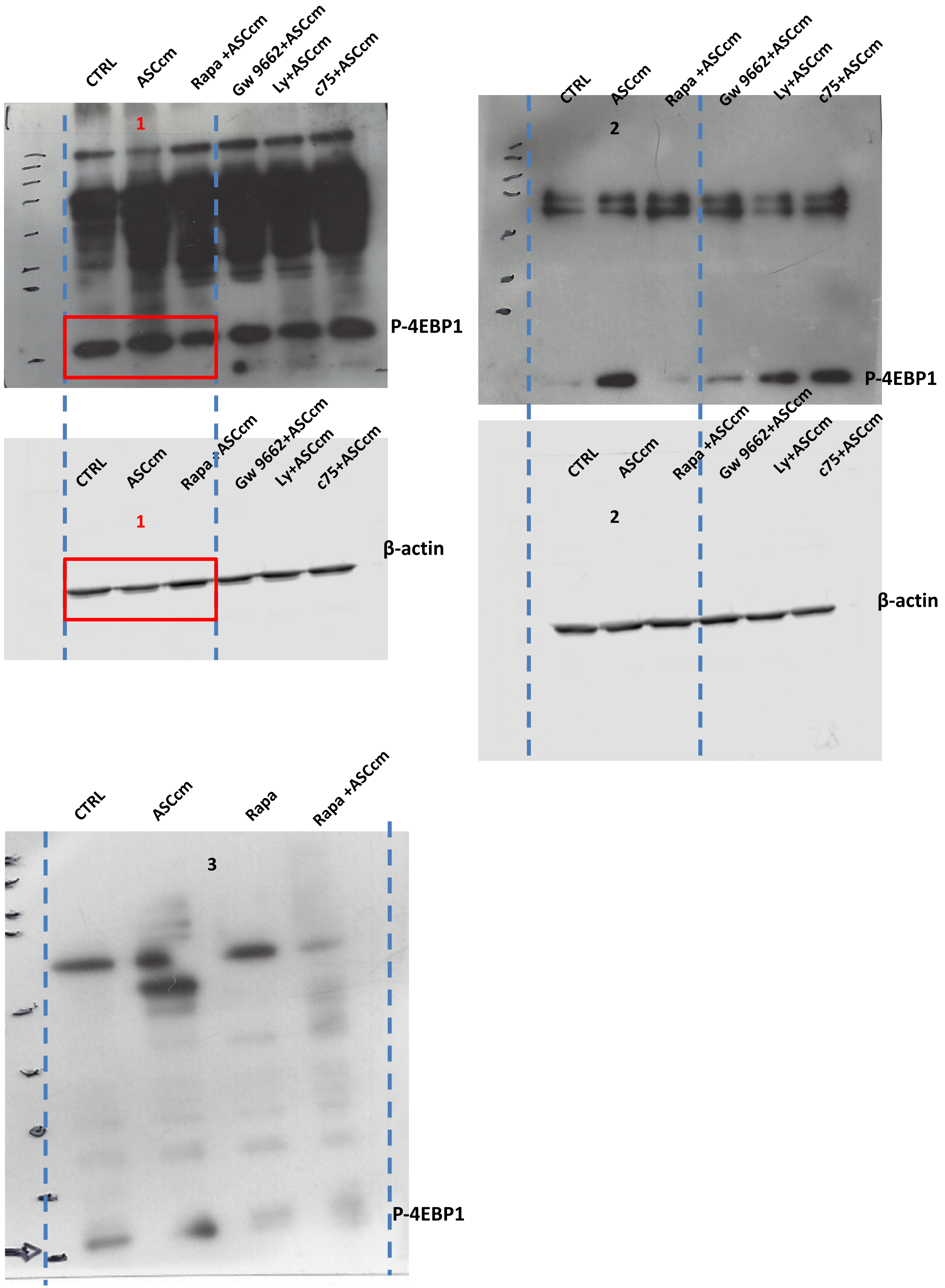

Figure 3B

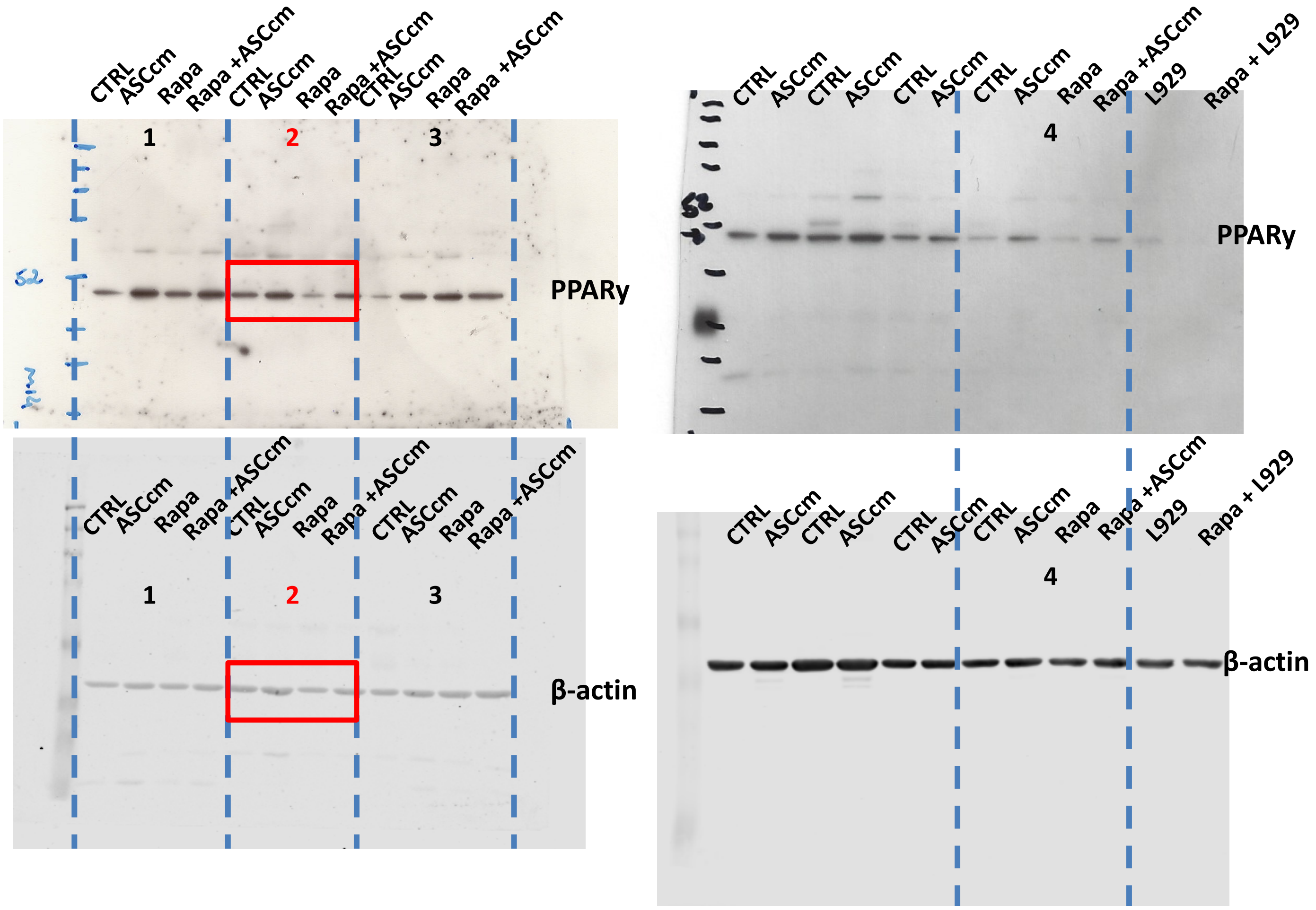

Figure 3C

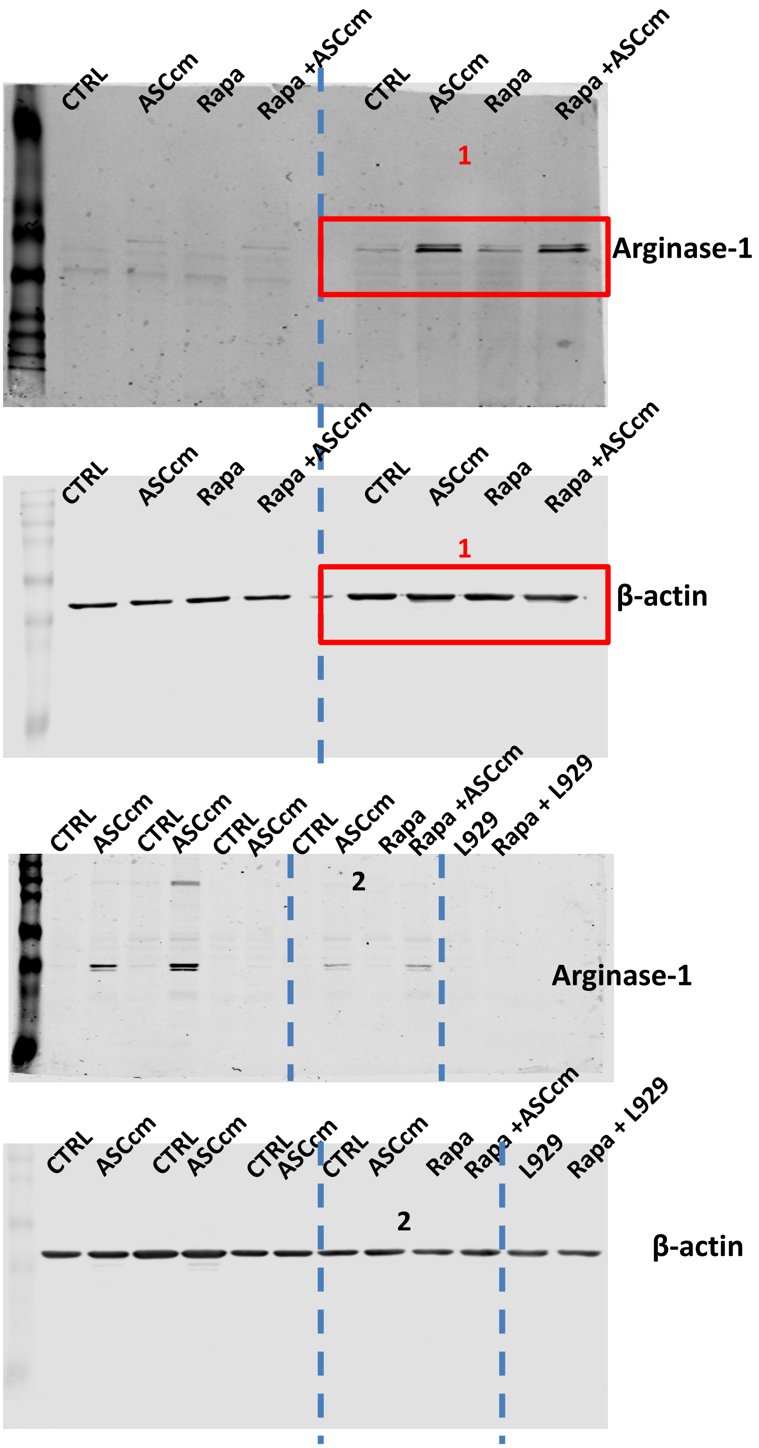
CTRL
ASCcm
Rapa
Rapa +ASCcm
CTRL
ASCcm
Rapa
Rapa +ASCcm
1
Arginase-1
CTRL
ASCcm
Rapa
Rapa +ASCcm
CTRL
ASCcm
Rapa
Rapa +ASCcm
1
β-actin
CTRL
ASCcm
CTRL
ASCcm
CTRL
ASCcm
CTRL
ASCcm
Rapa
Rapa +ASCcm
L929
Rapa + L929
2
Arginase-1
CTRL
ASCcm
CTRL
ASCcm
CTRL
ASCcm
CTRL
ASCcm
Rapa
Rapa +ASCcm
L929
Rapa + L929
2
β-actin

Figure 3C

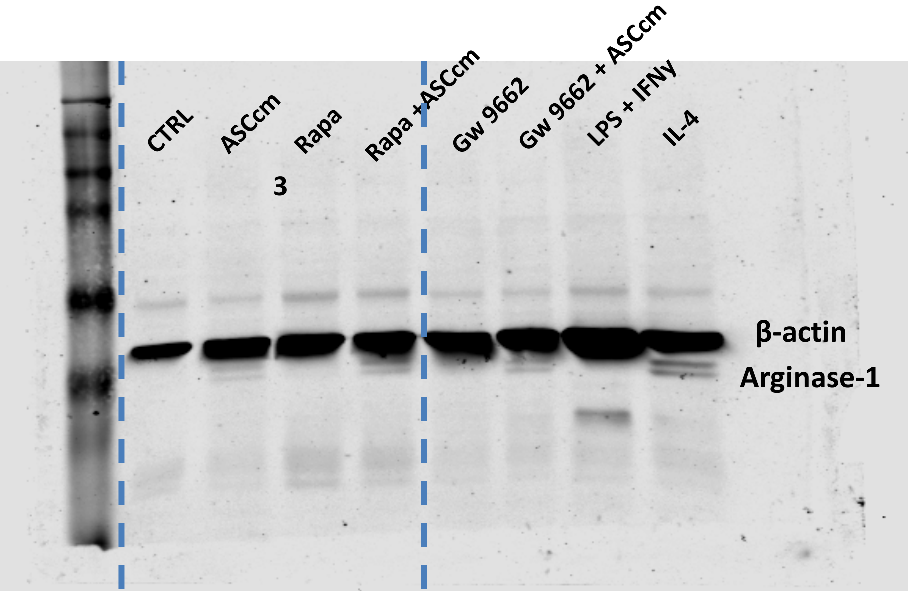
CTRL
ASCcm
Rapa
Rapa +ASCcm
Gw 9662
Gw 9662 + ASCcm
LPS + IFNγ
IL-4
3
β-actin
Arginase-1

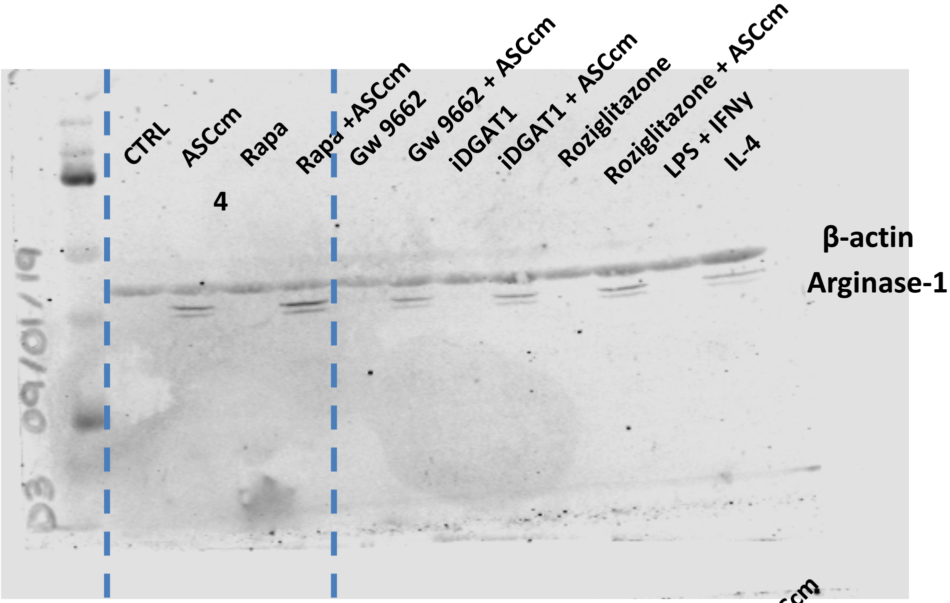
CTRL
ASCcm
Rapa
Rapa +ASCcm
Gw 9662
Gw 9662 + ASCcm
iDGAT1
iDGAT1 + ASCcm
Rozigliitazone
Roziglitazone + ASCcm
LPS + IFNγ
IL-4
4
β-actin
Arginase-1

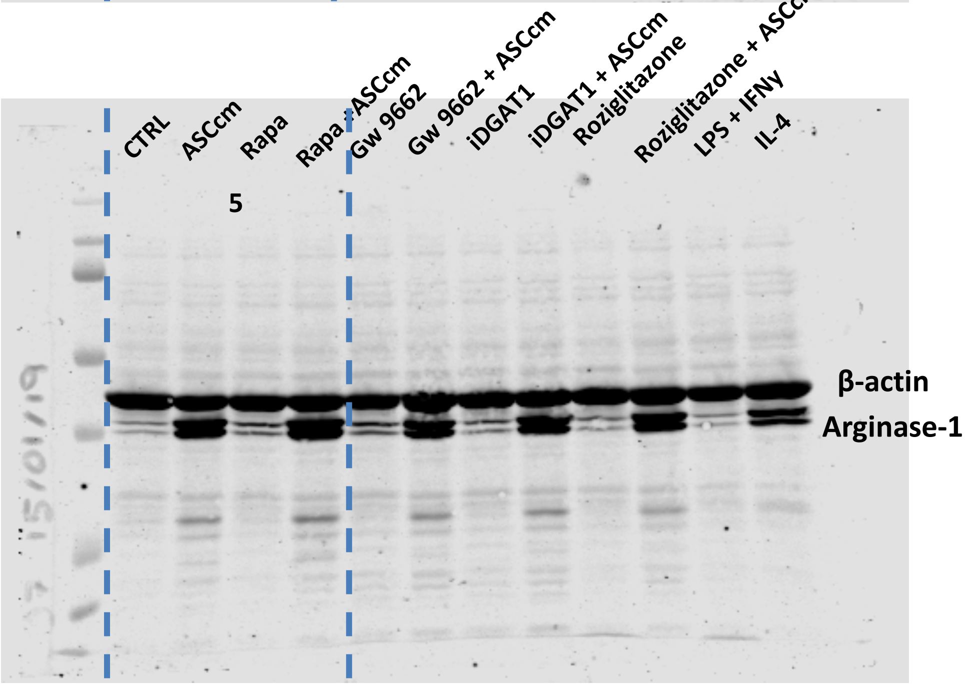
CTRL
ASCcm
Rapa
Rapa +ASCcm
Gw 9662
Gw 9662 + ASCcm
iDGAT1
iDGAT1 + ASCcm
Roziglitazone
Roziglitazone + ASCcm
LPS + IFNγ
IL-4
5
β-actin
Arginase-1

Figure 3D

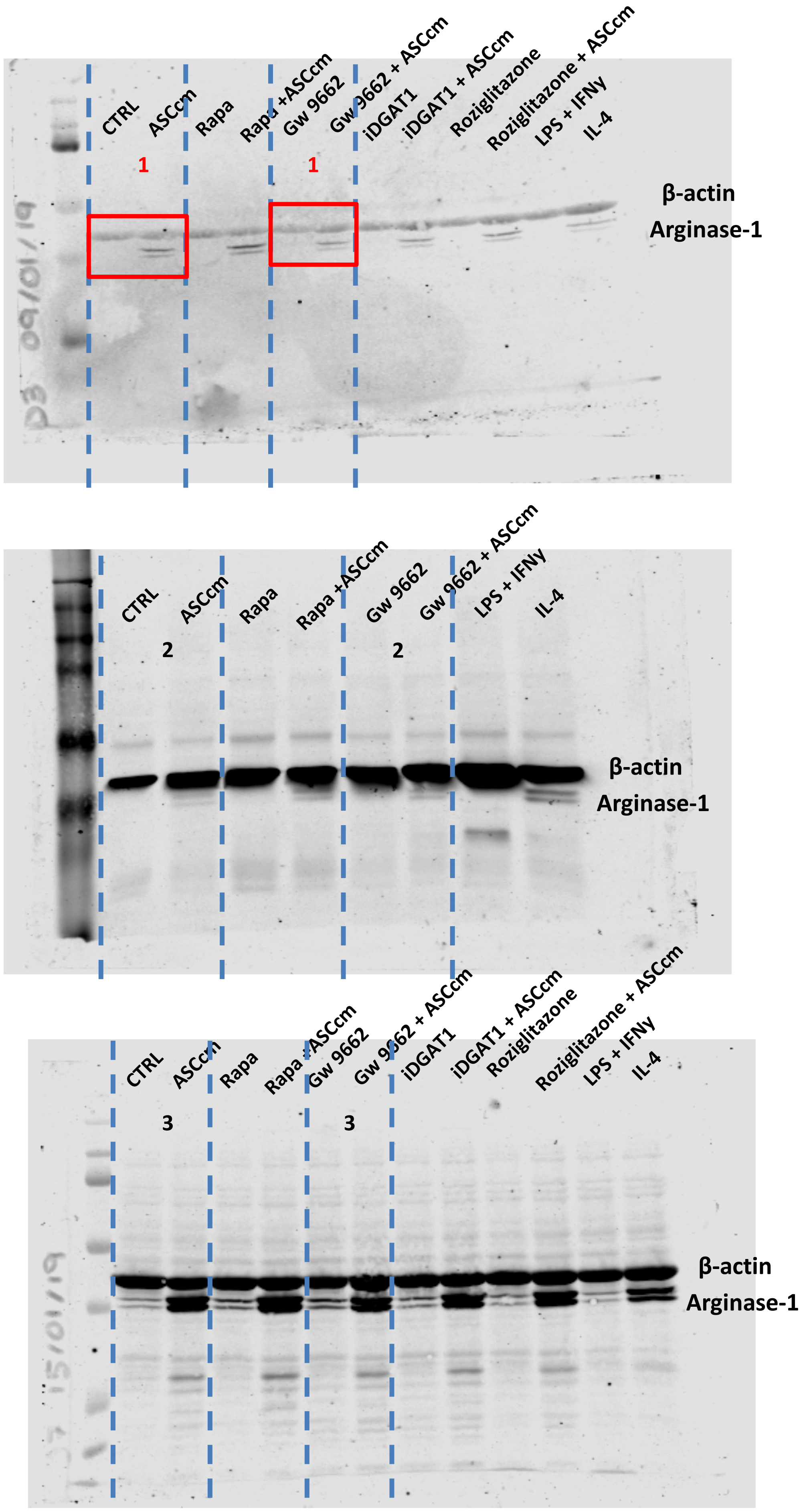
CTRL
ASCcm
Rapa
Rapa +ASCcm
Gw 9662
Gw 9662 + ASCcm
iDGAT1
iDGAT1 + ASCcm
Roziglitazone
Roziglitazone + ASCcm
LPS + IFNγ
IL-4
1
1
β-actin
Arginase-1
CTRL
ASCcm
Rapa
Rapa +ASCcm
Gw 9662
Gw 9662 + ASCcm
LPS + IFNγ
IL-4
2
2
β-actin
Arginase-1
CTRL
ASCcm
Rapa
Rapa +ASCcm
Gw 9662
Gw 9662 + ASCcm
iDGAT1
iDGAT1 + ASCcm
Roziglitazone
Roziglitazone + ASCcm
LPS + IFNγ
IL-4
3
3
β-actin
Arginase-1

Figure 3D

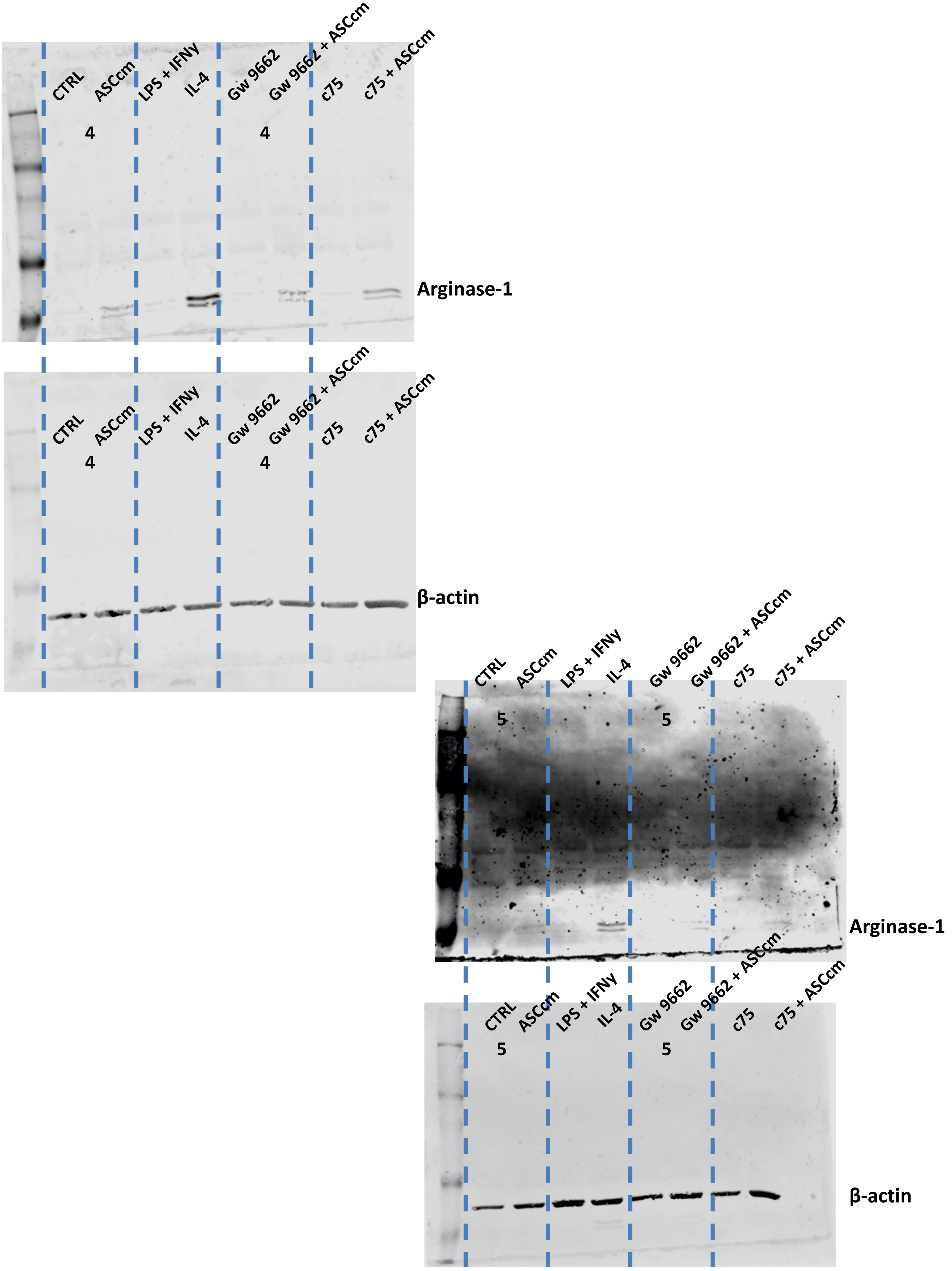

CTRL
ASCcm
LPS + IFNγ
IL-4
Gw 9662
Gw 9662 + ASCcm
c75
c75 + ASCcm
4
4
Arginase-1
CTRL
ASCcm
LPS + IFNγ
IL-4
Gw 9662
Gw 9662 + ASCcm
c75
c75 + ASCcm
4
4
β-actin
CTRL
ASCcm
LPS + IFNγ
IL-4
Gw 9662
Gw 9662 + ASCcm
c75
c75 + ASCcm
5
5
Arginase-1
CTRL
ASCcm
LPS + IFNγ
IL-4
Gw 9662
Gw 9662 + ASCcm
c75
c75 + ASCcm
5
5
β-actin

Figure 6A

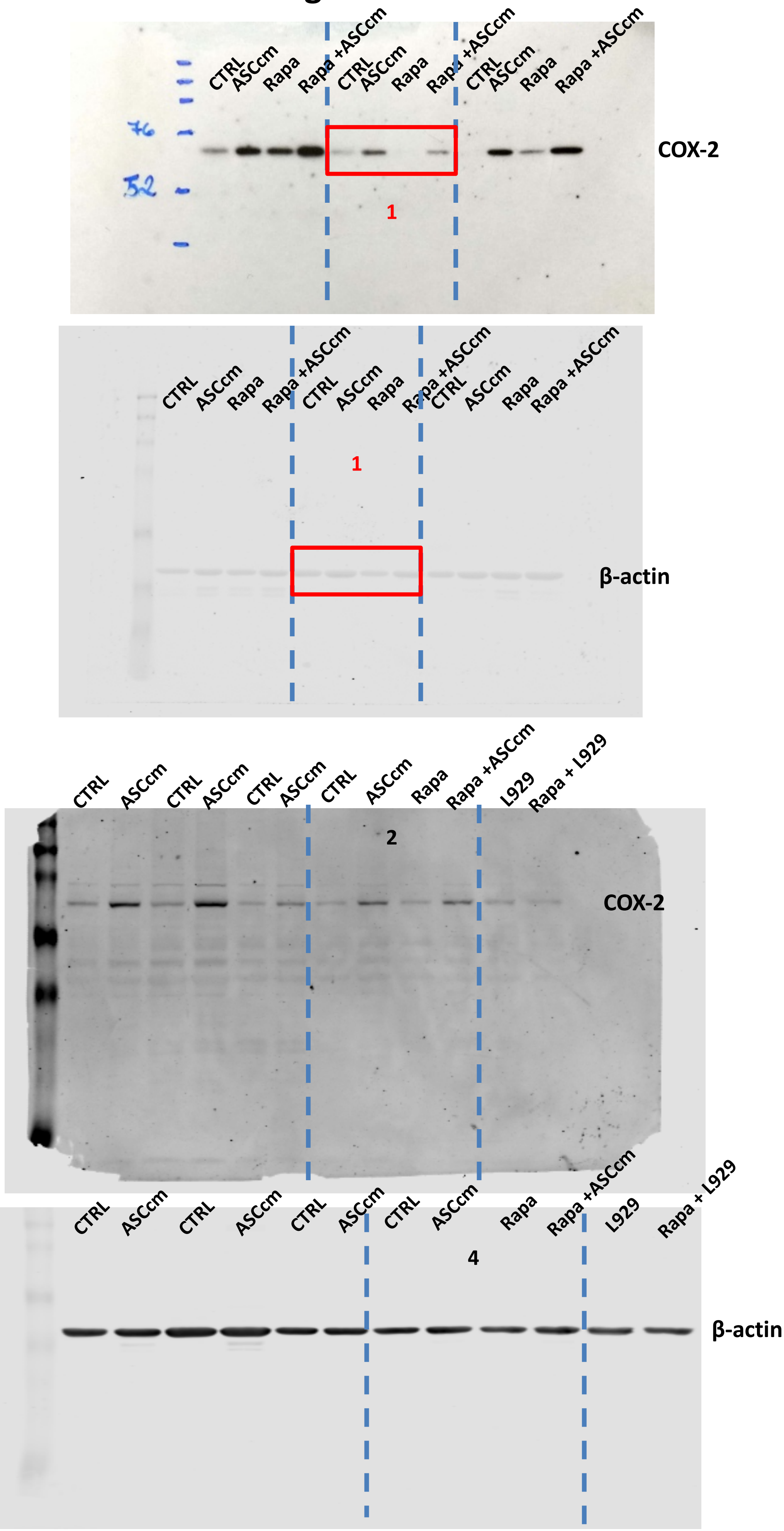
CTRL
ASCcm
Rapa
Rapa +ASCcm
76
52
1
COX-2
β-actin
2
L929
Rapa + L929
4

Figure 6A

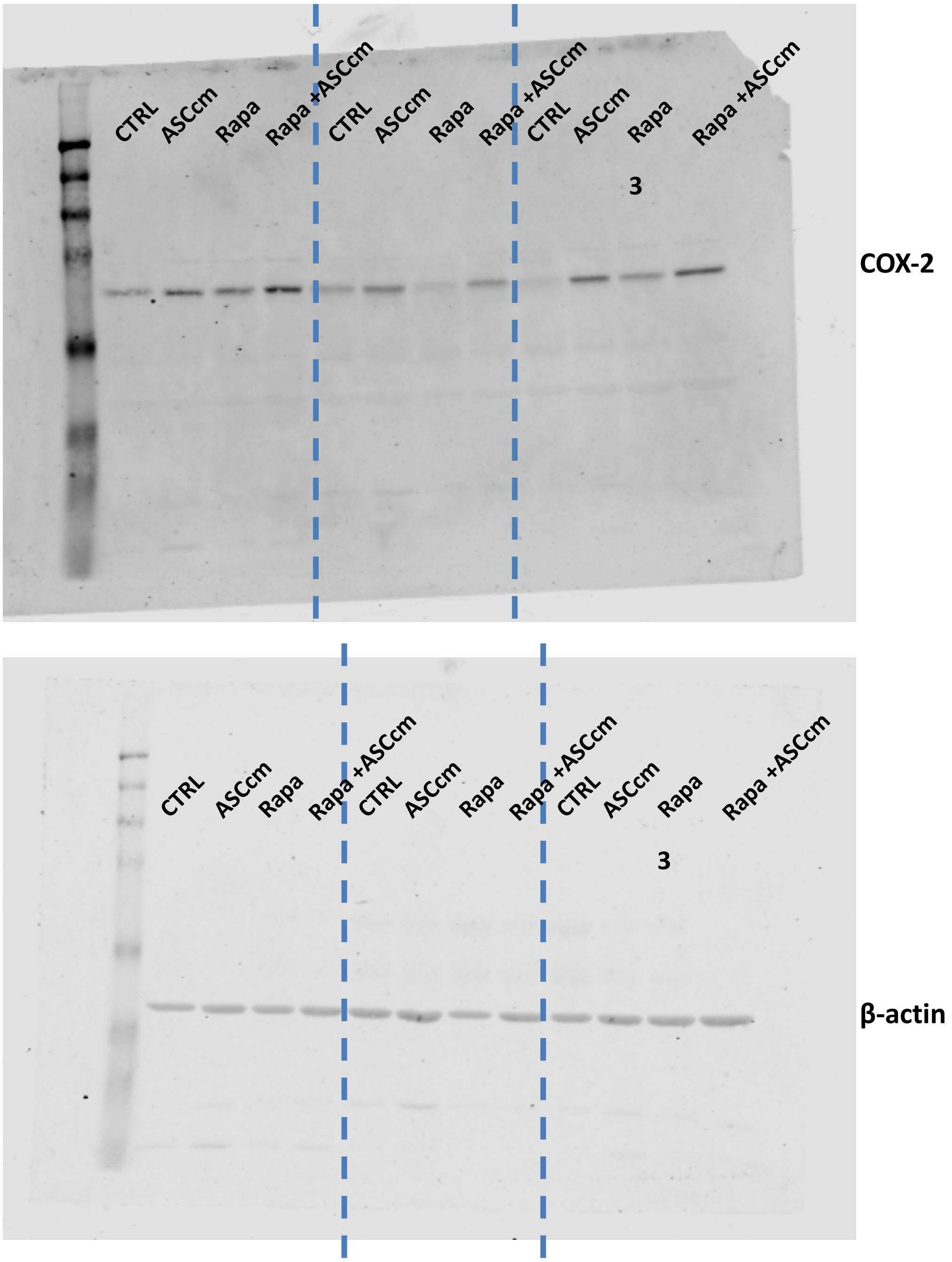

CTRL
ASCcm
Rapa
Rapa +ASCcm
CTRL
ASCcm
Rapa
Rapa +ASCcm
CTRL
ASCcm
Rapa
Rapa +ASCcm
3
COX-2
CTRL
ASCcm
Rapa
Rapa +ASCcm
CTRL
ASCcm
Rapa
Rapa +ASCcm
CTRL
ASCcm
Rapa
Rapa +ASCcm
3
β-actin

Figure 6A

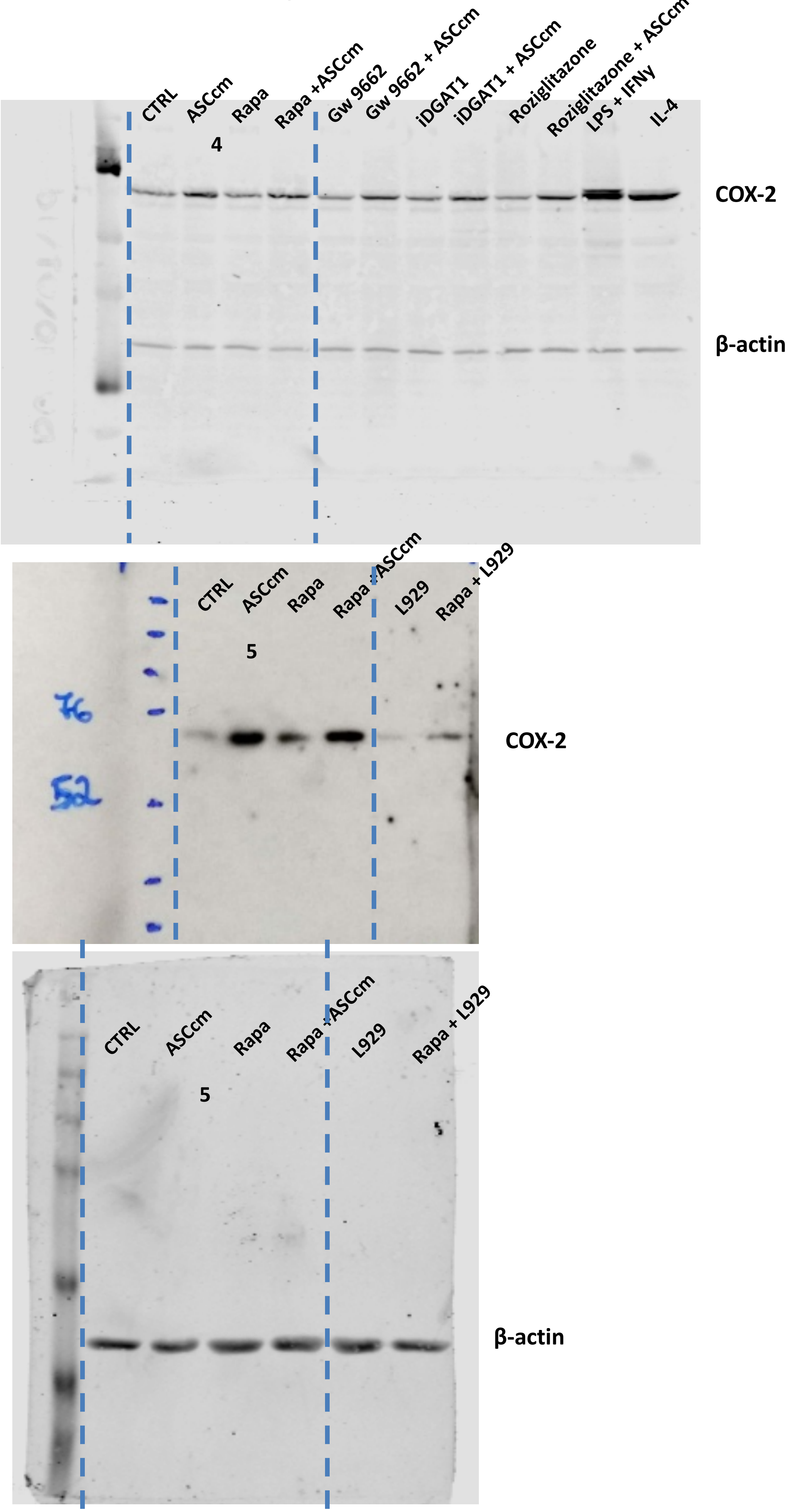
CTRL
ASCcm
Rapa
Rapa +ASCcm
Gw 9662
Gw 9662 + ASCcm
iDGAT1
iDGAT1 + ASCcm
Roziglitazone
Roziglitazone + ASCcm
LPS + IFNγ
IL-4
4
COX-2
β-actin
CTRL
ASCcm
Rapa
Rapa +ASCcm
L929
Rapa + L929
5
76
52
COX-2
CTRL
ASCcm
Rapa
Rapa +ASCcm
L929
Rapa + L929
5
β-actin

Figure 6B

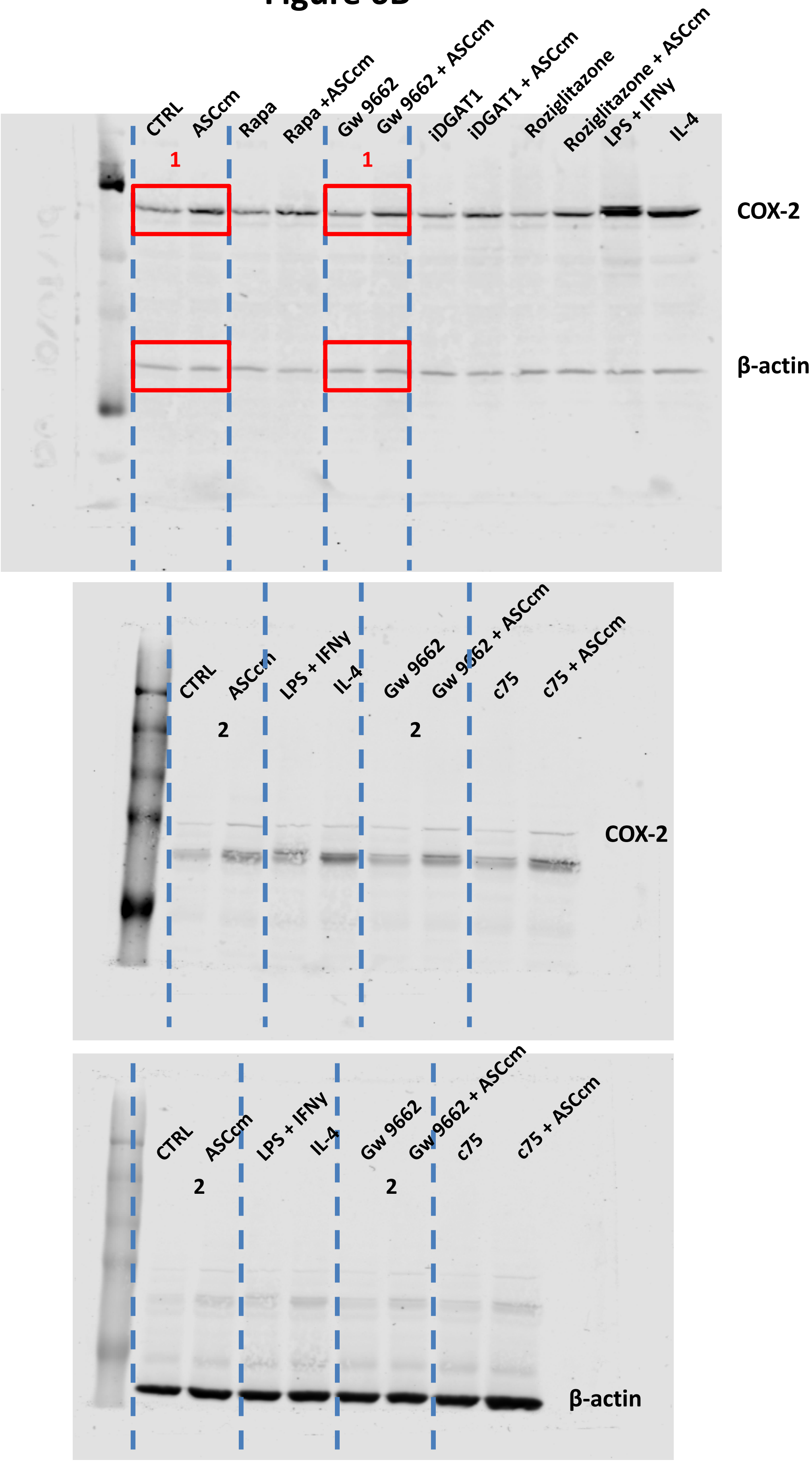
CTRL
ASCcm
Rapa
Rapa +ASCcm
Gw 9662
Gw 9662 + ASCcm
iDGAT1
iDGAT1 + ASCcm
Roziglitazone
Roziglitazone + ASCcm
LPS + IFNγ
IL-4
1
1
COX-2
β-actin
CTRL
ASCcm
LPS + IFNγ
IL-4
Gw 9662
Gw 9662 + ASCcm
c75
c75 + ASCcm
2
2
COX-2
CTRL
ASCcm
LPS + IFNγ
IL-4
Gw 9662
Gw 9662 + ASCcm
c75
c75 + ASCcm
2
2
β-actin

Figure 6B

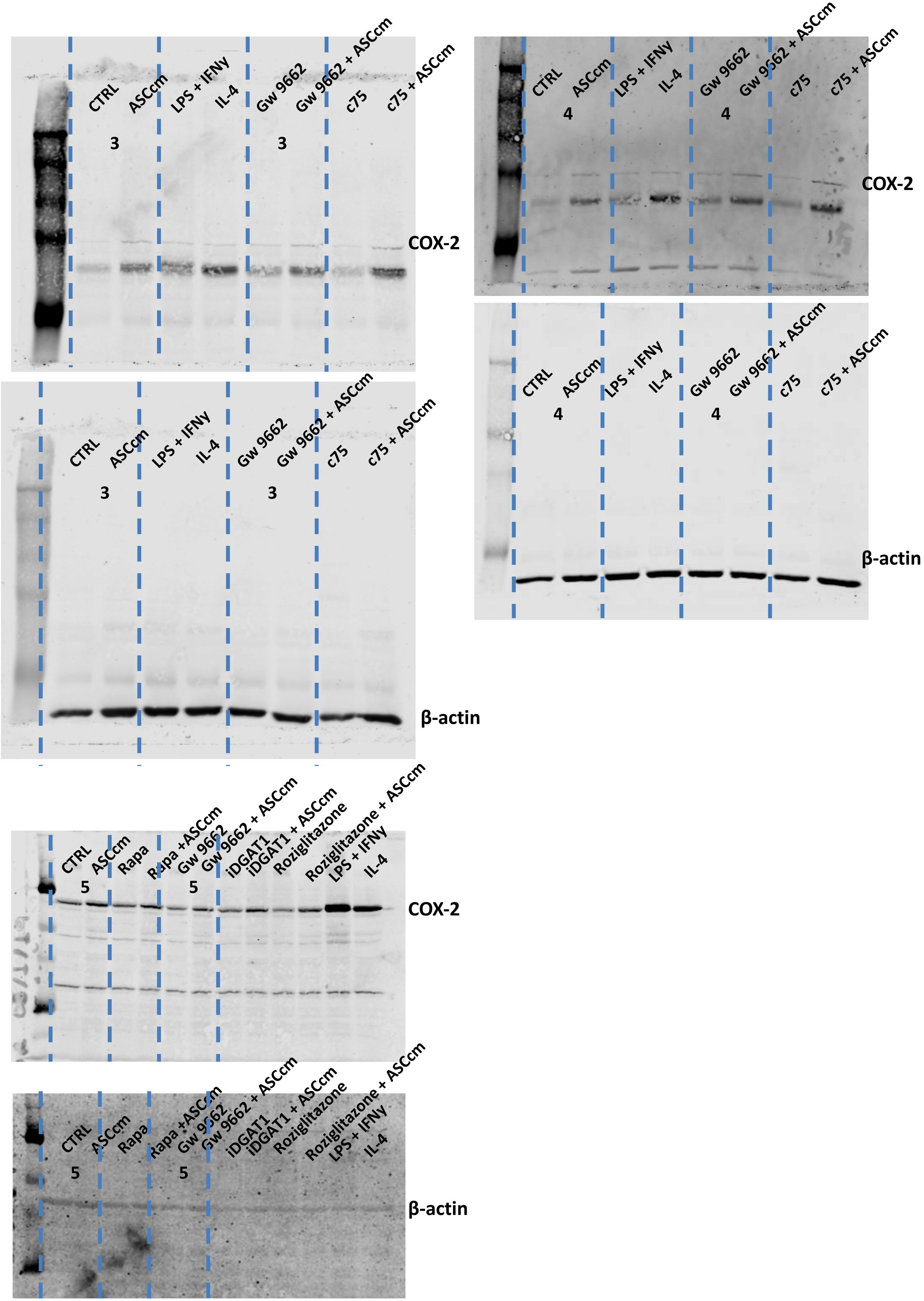
CTRL
ASCcm
LPS + IFNγ
IL-4
Gw 9662
Gw 9662 + ASCcm
c75
c75 + ASCcm
3
COX-2
β-actin
4
Rapa
Rapa +ASCcm
iDGAT1
iDGAT1 + ASCcm
Roziglitazone
Roziglitazone + ASCcm
5
